# Infrared Multiple Photon Dissociation Spectroscopy Confirms Reversible Water Activation in Mn^+^(H_2_O)_*n*_, *n* ≤ 8

**DOI:** 10.1021/acs.jpclett.2c00394

**Published:** 2022-04-07

**Authors:** Jakob Heller, Ethan M. Cunningham, Christian van der Linde, Milan Ončák, Martin K. Beyer

**Affiliations:** Institut für Ionenphysik und Angewandte Physik, Universität Innsbruck, Technikerstraße 25, 6020, Innsbruck, Austria

## Abstract

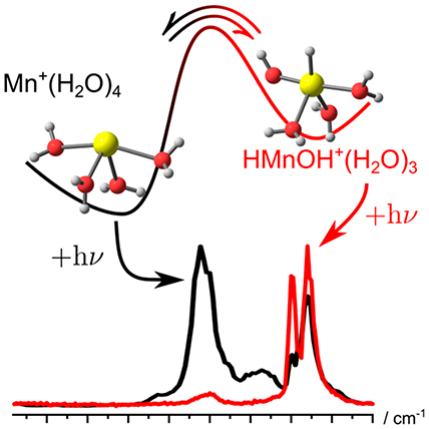

Controlled activation of water molecules is the key to efficient water splitting. Hydrated singly charged manganese ions Mn^+^(H_2_O)_*n*_ exhibit a size-dependent insertion reaction, which is probed by infrared multiple photon dissociation spectroscopy (IRMPD) and FT-ICR mass spectrometry. The noninserted isomer of Mn^+^(H_2_O)_4_ is formed directly in the laser vaporization ion source, while its inserted counterpart HMnOH^+^(H_2_O)_3_ is selectively prepared by gentle removal of water molecules from larger clusters. The IRMPD spectra in the O–H stretch region of both systems are markedly different, and correlate very well with quantum chemical calculations of the respective species at the CCSD(T)/aug-cc-pVDZ//BHandHLYP/aug-cc-pVDZ level of theory. The calculated potential energy surface for water loss from HMnOH^+^(H_2_O)_3_ shows that this cluster ion is metastable. During IRMPD, the system rearranges back to the noninserted Mn^+^(H_2_O)_3_ structure, indicating that the inserted structure requires stabilization by hydration. The studied system serves as an atomically defined single-atom redox-center for reversible metal insertion into the O–H bond, a key step in metal-centered water activation.

Water activation at metal centers is a crucial step in water splitting and formation of molecular hydrogen.^1−4^ Since actual (photo)electrocatalytic systems are extremely complex, model systems are needed to understand elementary reactions and key intermediates of water activation. It has been shown that hydrated metal ions in the gas phase are ideally suited for this purpose, as molecular-level mechanistic details can be probed in splendid isolation, without being obscured by counterions, aggregation, or other effects which can be difficult to control.^5^ As gas-phase species are normally more reactive than condensed-phase analogues, gas-phase experiments do not consider the complicated mechanisms in solution or at the surface; however, they provide the possibility to study elementary reactions under well-defined conditions. Hydrogen evolution on the ground state potential energy surface has been observed in mass spectrometric experiments in specific hydrated metal ions M^+^(H_2_O)_*n*_, with M = Mg,^6,7^ Al,^8,9^ and V.^10^ These reactions occur on a time scale of seconds, activated by blackbody radiation. H atom elimination from Mg^+^(H_2_O)_*n*_, which is experimentally observed for 16 ≤ *n* < 21, involves the coexistence of Mg^2+^ and a hydrated electron within the water cluster.^11−13^ Elimination of molecular hydrogen, however, is preceded by an intracluster proton transfer reaction converting M^+^(H_2_O)_*n*_ to HMOH^+^(H_2_O)_*n*−1_.^14−16^ D_2_O exchange^17−20^ and blackbody infrared radiative dissociation (BIRD)^21−27^ experiments on first row transition metal–water complexes, M^+^(H_2_O)_*n*_, M = Cr–Zn, suggested that also Mn^+^ doped water clusters undergo an intracluster redox reaction to form HMnOH^+^(H_2_O)_*n*−1_, without elimination of H or H_2_.^28^ The insertion reaction starts at around eight water molecules, in which Mn^+^(H_2_O)_8_ is converted to HMnOH^+^(H_2_O)_7_ under the influence of room temperature blackbody radiation.^28^ This water activation occurs in a similar size range for Al^+^(H_2_O)_*n*_ and V^+^(H_2_O)_*n*_, for which we recently probed the transition from M^+^(H_2_O)_*n*_ to HMOH^+^(H_2_O)_*n*-1_ by ultraviolet–visible (UV–vis) spectroscopy.^29,30^

Duncan and co-workers studied the singly hydrated species Mn^+^(H_2_O) and Mn^2+^(H_2_O) by infrared multiple photon photodissociation (IRMPD) spectroscopy using tagging with up to four Ar atoms.^31^ Metz and co-workers performed UV photodissociation on Mn^+^(H_2_O) and Mn^+^(D_2_O) in the 30000–35000 cm^–1^ region.^32^ The Garand group investigated the extent of charge transfer from OH^–^ to the metal center in MnOH^+^(H_2_O) by cryogenic ion infrared predissociation spectroscopy. The degree of charge transfer in MnOH^+^(H_2_O) was found to be smaller when compared to the later first row transition metals up to Cu.^33^ Up to three water molecules directly coordinate to the metal center in MnOH^+^(H_2_O)_*n*_, while the fourth occupies the second solvation shell.^34^ Nonpolar molecules such as CH_4_, as studied by Dryza and Bieske, coordinate with up to six molecules directly to Mn^+^, showing no evidence of significant bond activation.^6^

In our earlier H_2_O/D_2_O exchange experiments, we obtained indirect evidence that Mn^+^(H_2_O)_4_ is formed in the ion source, while HMnOH^+^(H_2_O)_3_ is formed from BIRD of larger clusters.^28^ The metal ion inserting into an O–H bond requires an extended hydrogen-bonded network since concerted proton transfer through a chain of water molecules lowers the activation energy, studied in detail for hydrated aluminum ions.^14,15^ As for aluminum, the insertion reaction with manganese seems to become efficient around *n* = 8, forming HMnOH^+^(H_2_O)_7_.^28^ To confirm these findings spectroscopically, we selectively prepare the two different classes of isomers of the [Mn(H_2_O)_4_]^+^ ion and perform infrared multiple photon dissociation (IRMPD)^35−40^ experiments in the O–H stretch and H–O–H bend region.

To this end, IRMPD spectra were recorded in the 1450–1950 cm^–1^ and 2250–4000 cm^–1^ region, with laser powers of between 20–32 and 50–180 mW, respectively. Temporal widths of both spectral regions are 9 and 12 ns, respectively. The ions were generated in a laser vaporization source^41,42^ and stored in an ICR cell either at room temperature or cooled to ≈87 K, in the latter case minimizing the influence of blackbody radiation.^43^ Cluster ions were irradiated with light from a tunable optical-parametric oscillator (OPO) system operated at a pulse frequency of 1000 Hz, which amounted to quasi-continuous irradiation on the time scale of the ICR experiment. The cluster size of interest was mass-selected, irradiated for 0.2–20 s (Table S1), and a mass spectrum was recorded. Irradiation time was adjusted to account for water binding energy, photon energy, and laser power, as to obtain sufficient photofragmentation of the parent ion. This procedure was repeated 15 times for each infrared wavenumber to improve the signal-to-noise ratio. Photon absorption led to evaporation of water molecules. The fragment intensity was quantified by mass spectrometry. Further experimental details are provided in the Supporting Information (SI).

From the theoretical chemistry perspective, transition metals are notoriously difficult to model, requiring multireference approaches for a correct description. These methods, however, are time-consuming for larger systems and require careful design of the active space able to describe the relevant part of the potential energy surface consistently. For this reason, we limited ourselves here to single-reference methods, namely density functional theory (DFT) with the BHandHLYP functional for optimization and the coupled cluster (CC) approach for single-point energy evaluation along with the aug-cc-pVDZ basis set. The methods were benchmarked against the multireference configuration interaction approach (MRCI) and other DFT functionals for Mn^+^(H_2_O)_2_, see Figure S3 and Tables S2,S3. The benchmark shows that DFT methods underestimate the stability of Mn^+^(H_2_O)_2_ in the septet spin multiplicity compared to the quintet, with BHandHLYP providing the best results among 11 of the tested DFT functionals. Infrared spectra were modeled by implementing Gaussian functions to the band positions, each with a full-width-half-maximum (fwhm) of 30 cm^–1^, using a scaling factor of 0.92 in the 2250–4000 cm^–1^ region to match the position of the free O–H band in the Mn^+^(H_2_O)_8_ cluster at ∼3700 cm^–1^. The unusually low scaling factor (0.96–1.00 is usually recommended for DFT functionals along with the aug-cc-pVDZ basis set^44^) provides results consistent with spectra of B3LYP and CCSD approaches with scaling factors of 0.96 and 0.95, respectively (Figure S5). In the 1450–1950 cm^–1^ region, a scaling factor of 0.96 was used for BHandHLYP (Figure S6) that reproduces well the experimental position of the OH bending vibration. Stabilization of the electronic wave function was performed prior to each DFT/CC calculation; DFT and CC calculations were performed using the Gaussian16 software package,^45^ while multireference calculations were performed using Molpro.^46,47^

IRMPD spectra of Mn^+^(H_2_O)_4_ prepared in three different ways were recorded; ions were either mass-selected directly after trapping in the ICR cell, or prepared by evaporating the Mn^+^(H_2_O)_8_ cluster down to four water molecules either by BIRD at room temperature for 20 s or IR laser irradiation for 3 s at 3200 cm^–1^. The latter procedure, however, proved very stressful for the crystal steering mechanics in the OPO system, and we stopped at 3000 cm^–1^ to prevent damage of the instrument. In addition, the IRMPD spectra of Mn^+^(H_2_O)_8_ were measured. Figure 1a–c presents the spectra at room temperature, Figure 1d–f at a temperature of ≈87 K. The corresponding spectra in the 1450–1950 cm^–1^ region are provided in the SI (Figure S2).

**Figure 1:**
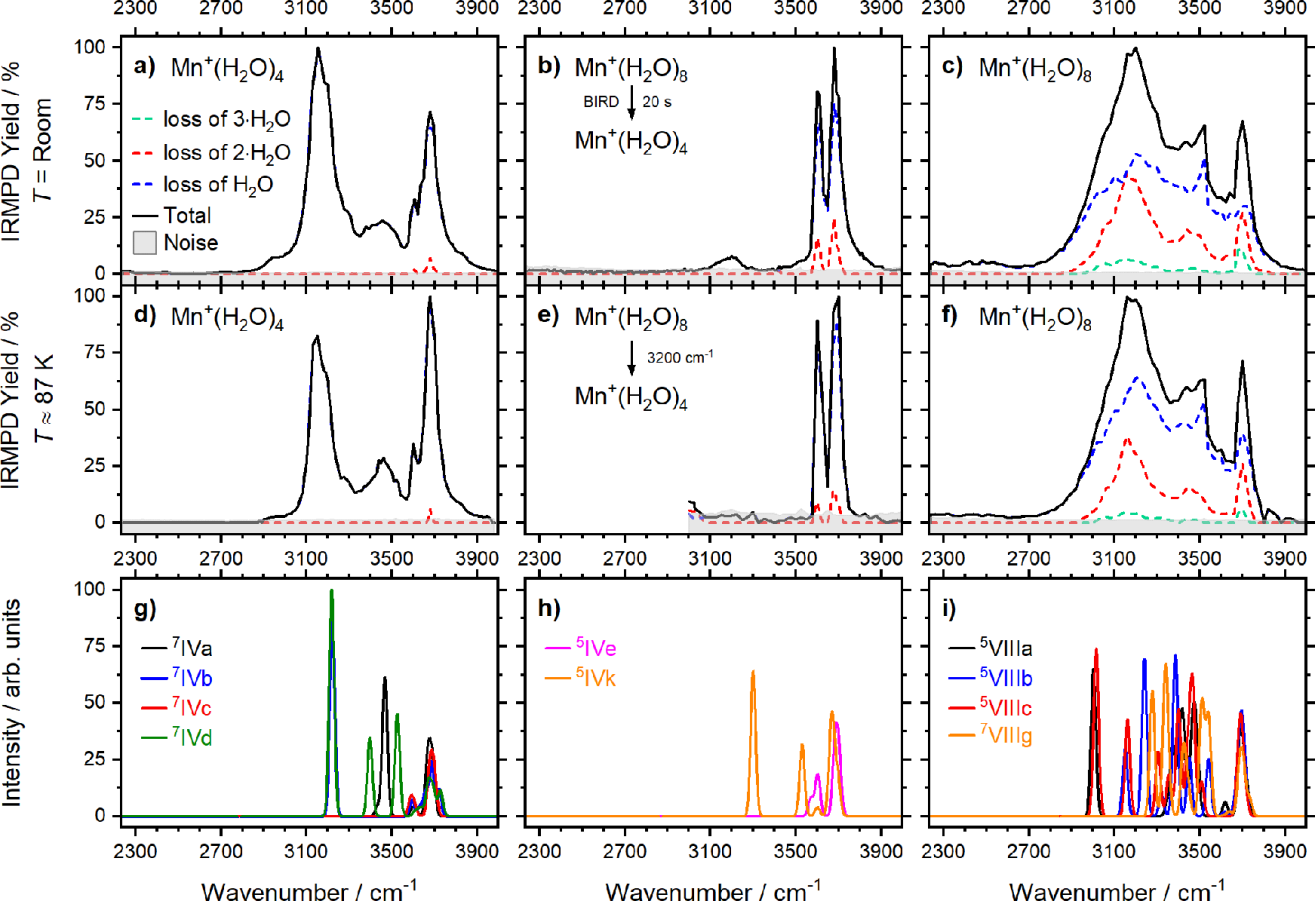
(a) Experimental IRMPD spectrum of Mn^+^(H_2_O)_4_ recorded at ≈298 K. (b) Experimental IRMPD spectrum of Mn^+^(H_2_O)_4_ recorded, and mass-selected, after Mn^+^(H_2_O)_8_ was subject to 20.0 s of blackbody infrared radiative dissociation (BIRD), at ≈298 K. (c) Experimental IRMPD spectrum of Mn^+^(H_2_O)_8_ recorded at *T* ≈ 298 K. (d) Experimental IRMPD spectrum of Mn^+^(H_2_O)_4_ recorded at ≈87 K. (e) Experimental IRMPD spectrum of Mn^+^(H_2_O)_4_ recorded, and mass-selected, after Mn^+^(H_2_O)_8_ was subject to 3.0 s of IRMPD at 3200 cm^–1^, at ≈87 K. (f) Experimental IRMPD spectrum of Mn^+^(H_2_O)_8_ recorded at ≈87 K. In panels g–i, infrared spectra were modeled at the BHandHLYP/aug-cc-pVDZ level with the scaling of 0.92; see Figure 2 for the respective isomers.

For Mn^+^(H_2_O)_4_ from the ion source, spectra at both temperatures present a strong band at 3150 cm^–1^ with a higher relative intensity at room temperature, a weaker band at 3460 cm^–1^, and an intense, structured band at 3680 cm^–1^ with a smaller maximum at 3600 cm^–1^ (Figure 1a,d). However, when Mn^+^(H_2_O)_4_ is formed from Mn^+^(H_2_O)_8_, the spectrum changes considerably (Figure 1b,e). There are only two prominent bands at 3600 and 3680 cm^–1^, with a contribution of a less intense band at 3190 cm^–1^ at room temperature. One could thus expect that two different classes of isomers are observed, Mn^+^(H_2_O)_4_ and HMnOH^+^(H_2_O)_3_, the latter formed by the conversion of Mn^+^(H_2_O)_8_ into HMnOH^+^(H_2_O)_7_ and water evaporation through BIRD or IR irradiation.

Figure 1c,f shows the IRMPD spectra of *n* = 8 recorded at room temperature and ≈87 K, respectively. Both spectra show an intense broad band centered at 3160 cm^–1^, along with bands at 3520 and 3700 cm^–1^. The band at 3160 cm^–1^ lies within the hydrogen bonding region, indicating a significant number of water molecules in the second solvation shell.

We used quantum chemical calculations to confirm the structural assignment (Figures 1g–i and 2; see Figures S8,S9 for other isomers). For *n* = 4, the most stable structure is Mn^+^(H_2_O)_4_ of septet spin multiplicity (Figure 2, isomers ^**7**^**IVa–d**) with a preferred coordination number of three. The most stable HMnOH^+^(H_2_O)_3_ isomer with the metal ion inserted into an O–H bond has quintet multiplicity and lies at 41 kJ mol^–1^ (^**5**^**IVe**), with a coordination number of five.

**Figure 2:**
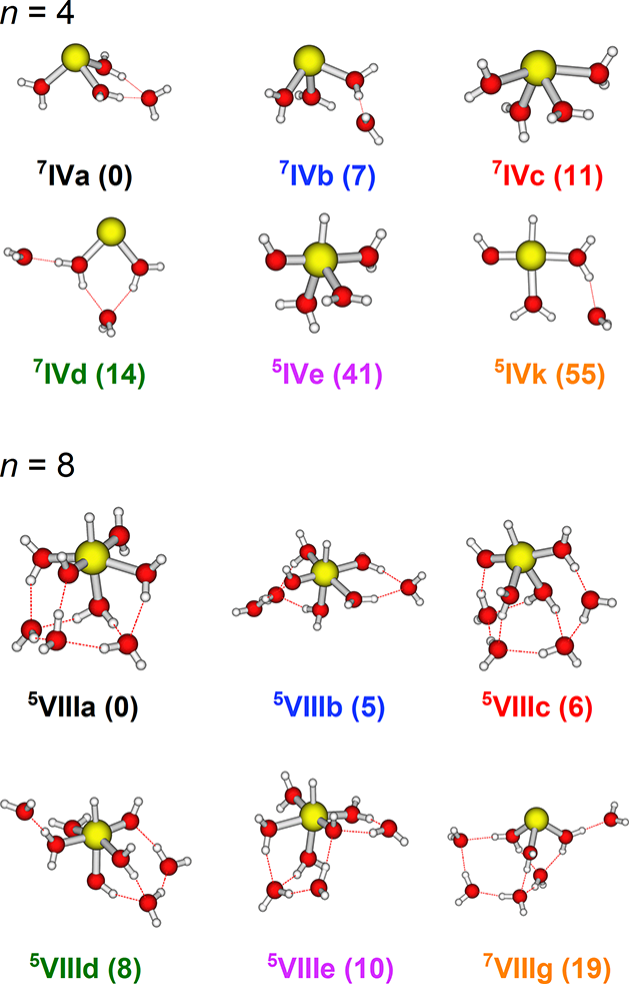
Selected low-energy structures of Mn^+^(H_2_O)_*n*_ and HMnOH^+^(H_2_O)_*n*−1_ for *n* = 4 and 8. Relative energies in kJ mol^–1^ were evaluated at the CCSD(T)/aug-cc-pVDZ//BHandHLYP/aug-cc-pVDZ level of theory. For further isomers, see Figures S8 and S9.

Analysis of calculated IR transitions shows that the spectra in Figure 1a,d originate mostly from Mn^+^(H_2_O)_4_. The strong band at 3150 cm^–1^ agrees with the simulated band at 3220 cm^–1^ in isomers ^**7**^**IVb** and ^**7**^**IVd,** the single-acceptor (SA) binding motif. A higher relative intensity at room temperature (Figure 1a) signifies a higher abundance of isomers with this motif. This effect has been observed before in M^+^(H_2_O)_*n*_ clusters, whereby at elevated temperatures entropic effects dominate enthalpic effects, that is, more SA (entropic) motifs are present.^48−50^ The weaker band at 3460 cm^–1^ agrees with the simulated band at 3470 cm^–1^ in isomer ^**7**^**IVa**, the double-acceptor (DA) motif. Finally, all ^**7**^**IVa–d** isomers might contribute to the band at 3680 cm^–1^ composed of free O–H stretches.

Spectra in Figure 1b,e reveal the signature of HMnOH^+^(H_2_O)_3_, namely isomer ^**5**^**IVe** with bands at ∼3600 and 3690 cm^–1^ corresponding to stretches of the O–H ligand and asymmetric water stretches. The band at ∼3190 cm^–1^ observed at room temperature could correspond to the SA motif in ^**5**^**IVk** at ∼3300 cm^–1^. No evidence is found for the presence of the noninserted isomers, as the characteristic band at 3470 cm^–1^ in ^**7**^**IVa** is not observed.

Kinetic experiments concluded that BIRD of larger clusters leads to 100% of HMnOH^+^(H_2_O)_3_ isomers, while ions from the source were composed of 80% Mn^+^(H_2_O)_4_ and 20% HMnOH^+^(H_2_O)_3_.^28^ However, source conditions may be different in the current study, since a new laser system was used. On the basis of the spectrum presented in Figure 1a, we cannot rule out a small contribution of inserted isomers, as the simulated spectra of ^**5**^**IVe** and ^**5**^**IVk** overlap with the experimental bands. The pronounced shoulder at 3600 cm^–1^ in Figure 1a is centered at the same position as the intense feature in Figure 1b, which would be consistent with the presence, albeit in lower abundance, of inserted isomers, similar to the earlier BIRD kinetic experiments.^28^ However, the shoulder is also in agreement with the free O–H bands in the noninserted isomers ^**7**^**IVa–d**, presented in Figure 1g. In any event, the striking differences between the spectra in Figure 1a,b clearly show that different structural isomers are probed.

The broad bands present in the *n* = 8 spectrum do not afford a clear structural assignment. Our calculations at the CCSD(T)/aug-cc-pVDZ//BHandHLYP/aug-cc-pVDZ level suggest that the inserted structure is slightly more stable (Figure 2), in quantitative agreement with BIRD experiments,^28^ where an energy difference of 21 ± 10 kJ mol^–1^ was reported. The putative global minimum structure (^**5**^**VIIIa**) is an inserted, quintet isomer with a coordination number of 6. The five lowest-lying isomers found are all inserted, 5- or 6-fold coordinated with quintet multiplicity lying within 10 kJ mol^–1^. The noninserted septet isomer ^**7**^**VIIIg** lies 19 kJ mol^–1^ higher in energy and is 3-fold coordinated. The simulated spectra shown in Figure 1i are heavily congested, showing many different bands, in general agreement with the experiment.

The qualitative differences in the spectra presented in Figure 1 panels a and b, along with the agreement with the simulated spectra, present strong evidence for the presence of inserted isomers in the spectrum in Figure 1b, produced via evaporation of water from HMnOH^+^(H_2_O)_7_. Note that the most stable inserted isomer for *n* = 4, ^**5**^**IVe**, is calculated to lie 41 kJ mol^–1^ above the lowest-lying Mn^+^(H_2_O)_4_ isomer ^**7**^**IVa**. This implies that we have prepared this ion as a long-lived, metastable isomer.

In Figure 3, we analyze the stability and dissociation pathways for ^**7**^**IVa** and ^**5**^**IVe** employing quantum chemistry. For ^**7**^**IVa**, one can see that the cluster might rearrange within the septet manifold already at low energies, for example, with a barrier of 16 kJ mol^–1^ through **TS3** to form ^**7**^**IVc**, in agreement with the presence of several isomers in the experimental spectrum (Figure 1a,d). When the cluster is irradiated with several IR photons, water evaporation is predicted as the most probable channel, with a reaction energy of 62 kJ mol^–1^. Rearrangement to the inserted structures faces a barrier of 103 kJ mol^–1^ and can be ruled out.

**Figure 3:**
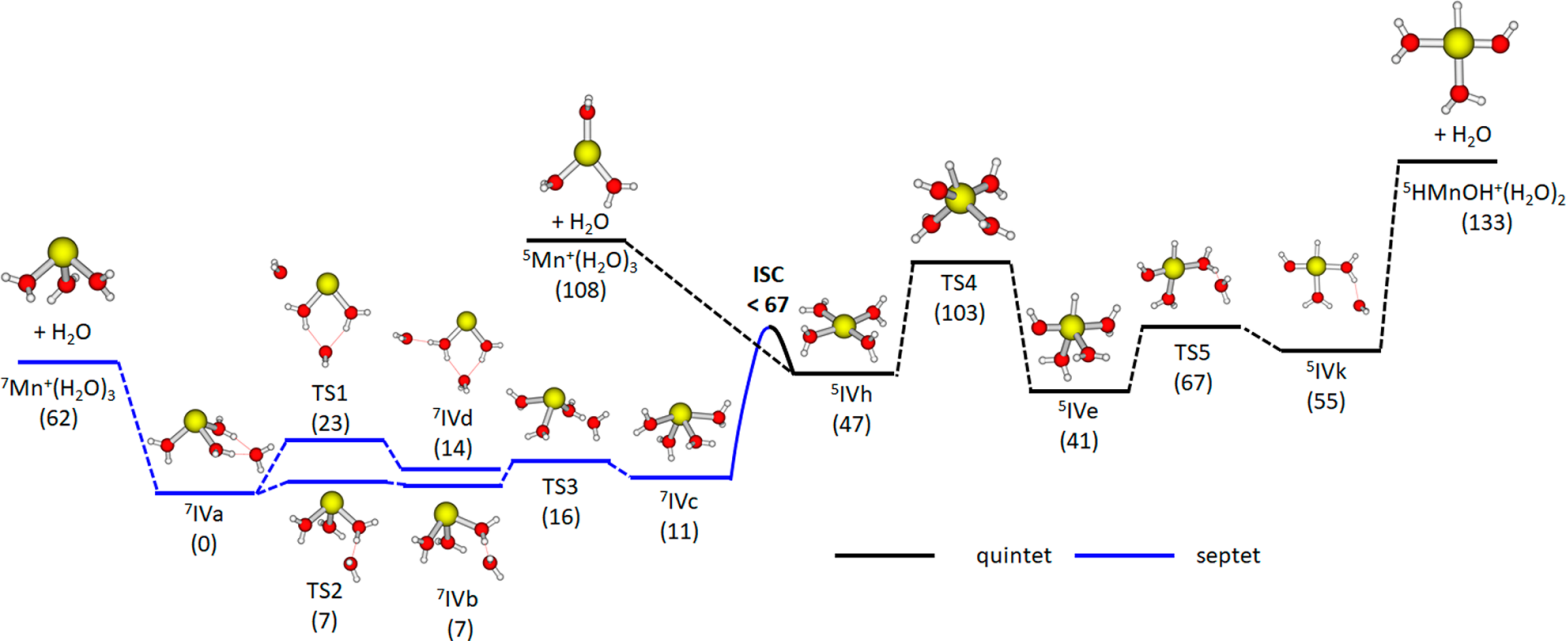
Simplified reaction pathway for water loss from the Mn^+^(H_2_O)_4_ cluster. Relative energies in kJ mol^–1^ were evaluated at the CCSD(T)/aug-cc-pVDZ//BHandHLYP/aug-cc-pVDZ level of theory. The energy of intersystem crossing (ISC) is estimated through interpolation (Figure S10). Water might dissociate from all ^**7**^**IVa–e** isomers (not shown for clarity).

The inserted isomer HMnOH^+^(H_2_O)_3_, which can be selectively produced via BIRD or IR irradiation from HMnOH^+^(H_2_O)_7_, in turn may rearrange within the quintet manifold using the energy present in the cluster upon photon absorption, for example through **TS5**. Water evaporation to form HMnOH^+^(H_2_O)_2_, however, lies very high in energy with a reaction energy of 92 kJ mol^–1^ when starting from ^**5**^**IVe**. In contrast, proton transfer to form Mn^+^(H_2_O)_4_ in the quintet multiplicity only requires an activation energy of 62 kJ mol^–1^ (**TS4**), resulting in ^**5**^**IVh**. The intersystem crossing (ISC) from quintet to septet spin multiplicity should be easily surmounted. We estimate an upper bound of the minimum energy crossing point by interpolation between isomers ^**5**^**IVh** and ^**7**^**IVc** in internal coordinates (see Figure S10). The point at which both curves cross lies at 26 kJ mol^–1^ with respect to ^**5**^**IVe**, well below the energy of **TS4**. Therefore, the system might switch to septet spin multiplicity and evaporate a water molecule. In principle, water evaporation might take place directly from ^**5**^**IVh**, but this requires at least 41 kJ mol^–1^ more than reaching the ISC point, making this pathway less probable. The calculated reaction paths are in full agreement with the previous D_2_O exchange experiments where it was observed that for *n* = 4, one hydrogen atom cannot be exchanged, Mn^+^(DHO)(D_2_O)_3_, while for *n* = 3, Mn^+^(D_2_O)_3_ is formed.^28^ Calculations in Figure 3 clearly show that between *n* = 4 and 3, intersystem crossing from a quintet inserted structure HMnOH^+^(H_2_O)_3_ to a septet noninserted structure Mn^+^(H_2_O)_4_ is the preferred energetic pathway, when compared to water evaporation. The difference in cluster structure for *n* = 4 and *n* = 3 also explains experimentally observed differences in reactivity with NO.^28^ Mn(I), as present in the *n* = 3 species, is more reactive against NO than Mn(III), which is present in the *n* ≥ 4 species HMnOH^+^(H_2_O)_*n*-1_ in the reactivity experiment.

Finally, the direct evidence for the presence of the Mn–H bond would be the Mn–H stretching frequency revealed in the IR spectrum, which we recently identified in HAlOH^+^(H_2_O)_*n*-1_.^16^ Benchmarking of theoretical methods shows that BHandHLYP and B3LYP predict the Mn–H vibrational position to be red and blue-shifted, respectively, when compared to CCSD (Tables S4 and S5). On the basis of our calculations for *n* = 1–4, we estimate an unscaled Mn–H frequency of ∼1670 cm^–1^ for ^**5**^**IVe** at the CCSD level, i.e., about 1590 cm^–1^ using a scaling factor of 0.95, possibly coinciding with the H_2_O scissoring vibration band at 1580–1680 cm^–1^. Another issue making observation of the Mn–H bond IR signal complicated is its low intensity compared to the water scissoring by a factor of about 3–40 for *n* = 2, depending on the functional used (Table S6). Indeed, the Mn–H signal was not unambiguously observed in our experiment (Figure S2), although a small band at 1560 cm^–1^ lies close to our prediction of the Mn–H frequency position.

We have confirmed spectroscopically that Mn^+^(H_2_O)_*n*_ undergoes an insertion reaction forming HMnOH^+^(H_2_O)_*n*-1_, as indirectly inferred from our D_2_O exchange experiments. Our quantum chemical calculations show that the inserted structure is energetically preferred only for larger clusters, but experiment shows that the system remains trapped in the inserted geometry during water evaporation, down to *n* = 4. H_2_O evaporation from the inserted structure is very energetically demanding. The cluster rather rearranges to the noninserted geometry, which is associated with a change in spin multiplicity from quintet to septet. These subtle rearrangements involving redox reactions and water activation as well as deactivation, which depend sensitively on the coordination of the metal center, illustrate the complexity of electrochemical water splitting. The studied system serves as an atomically defined example of a reversible single-atom redox-center for metal insertion into the O–H bond, characterized by infrared spectroscopy and quantum chemistry with atomic precision, with implications for hydrogen fuel production, as well as light-harvesting mechanisms which underpin photochemical production of atomic and molecular hydrogen.

## Supporting Information Available

Experimental details; supplementary experimental results, representative mass spectra, IRMPD spectra in the 1450–1900 cm^–1^ region; supplementary calculations, benchmarking of the computational approach, additional Mn^+^(H_2_O)_4,8_ cluster structures and simulated spectra, calculated crossing between the quintet and septet state in Mn^+^(H_2_O)_4_; Cartesian coordinates and energies of all calculated structures; calculated IR transitions (PDF)
Ion intensities, laser power and IRMPD yields used to generate Figure 1 and Figure S2 (XLSX)
