# Supplementary material for: Infrared Multiple Photon Dissociation Spectroscopy Confirms Reversible Water Activation in Mn+(H2O)n, n ≤ 8
Source: J Phys Chem Lett. 2022 Apr 7;13(14):3269–75. doi: 10.1021/acs.jpclett.2c00394 (PMC9014459; doi:10.1021/acs.jpclett.2c00394)
Supplement: Supplementary file 1 — jz2c00394_si_001.pdf [file jz2c00394_si_001.pdf]

Supporting Information:

Infrared Multiple Photon Dissociation Spectroscopy Confirms Reversible Water Activation in $Mn^+(H_2O)_n$, $n \leq 8$

Jakob Heller, Ethan M. Cunningham, Christian van der Linde, Milan167Ončák,* Martin K. Beyer*

Institut für Ionenphysik und Angewandte Physik, Universität Innsbruck, Technikerstraße 25, 6020 Innsbruck, Austria

Corresponding authors: milan.oncak@uibk.ac.at; martin.beyer@uibk.ac.at

Contents

Experimental Details 2

Computational Data 7

Calculated Structures and IR Spectra 13

References 29

Experimental Details

All experimental measurements were performed on a modified 4.7 Tesla Bruker/Spectrospin FT-ICR CMS47X mass spectrometer[1–5] equipped with a Bruker infinity cell[6] and laser vaporisation source.[7,8] Briefly, a frequency-doubled Litron Nano S 60-30 Nd:YAG laser (532 nm, 5 mJ/pulse, 30 Hz) is focussed onto a rotating solid disc of manganese producing a plasma. This plasma is then entrained into a pulse of the desired gas mixture (H_2O in helium) produced *via* a homebuilt piezoelectric valve. The ensuing pulse is cooled *via* supersonic jet expansion into the source chamber. The gas pulse traverses through a skimmer (forming the molecular beam), and ions are guided *via* an electrostatic lens set-up into the center of the ICR cell. Ions are then stored and mass-selected within the 4.7 T magnetic field[9] under ultra-high vacuum conditions (*ca.* 5 x 10^{-10} mbar). The ICR cell is surrounded by a copper jacket, whereby the temperature of the cell can be controlled and cooled to *ca.* 87 K *via* liquid nitrogen,[10,11] minimising the effects of black body infrared radiative dissociation (BIRD).[12–17] In each case *either* the measurements were recorded at room temperature, or cooling of the ICR cell *via* liquid nitrogen was employed.

On the opposite side of the magnet, the output radiation of a tunable IR OPO laser system (EKSPLA NT277/273-XIR) is aligned into the cell through a CaF_2 window.[18] Absorption of infrared photons, leading to photodissociation events are measured *via* mass spectroscopy.[19] Monitoring the precursor and fragment abundance channels as a function of wavenumber yields the infrared spectrum of the complex of interest. Infrared spectra were recorded in i) the 2250–4000 cm^{-1} wavelength region, probing in the O–H symmetric and asymmetric stretching region, and ii) the 1450–1950 cm^{-1} region, probing the water bend and Mn–H stretch. Specific details on the laser setup can be found in previous publications.[18,20] Infrared spectra of size-selected clusters was recorded *via* action spectroscopy, reaction 1:

$$\mathrm{Mn}^{+}(\mathrm{H_2O})_n + m\,h\nu \rightarrow \mathrm{Mn}^{+}(\mathrm{H_2O})_{n-x} + x(\mathrm{H_2O}) \quad (1)$$

Typical irradiation times are between 0.2–20.0 s at 1000 Hz repetition rate. The normalized IRMPD Yield is calculated from the precursor ion and BIRD-corrected fragment ion intensities along with considerations given to the laser power. First, ion intensities are normalized so that the precursor ion intensity is 100%. In the next step, the fragment intensities are BIRD corrected by subtracting the fragment intensities form a control experiment without laser irradiation, but identical residence time in the ICR cell. IRMPD yields are then calculated as (sum over BIRD corrected fragment intensities)/(sum over BIRD corrected fragment intensities and precursor intensity)/(laser power) and re-normalized for graphical display so that the maximum IRMPD yield in the spectrum is 100%.

The laser power is measured after every mass spectrum to account for small fluctuations. The laser power drops in the range 3500 – 3520 cm^{-1}. Due to the complex kinetics of the IRMPD process, this may lead to small artifacts in the IRMPD yield at these wavelengths even after power correction.

In conjunction with the IRMPD measurements, BIRD experiments were also performed on the $Mn^+(H_2O)_8$ complex. Figure S1a shows a typical mass spectrum recorded during an experimental run, presenting $Mn^+(H_2O)_n$ clusters, n = 4–15. Figure S1a-e presents a sequence of mass spectra used to record the IRMPD spectrum presented in Figure 1b in the main article. First, the $Mn^+(H_2O)_8$ complex is mass-selected (Figure S1b), and is heated by room temperature black-body radiation from the ICR cell walls for 20 s (Figure S1c). The $[Mn,(H_2O)_4]^+$ complex is mass-selected (Figure S1d) and subject to IR radiation at 3680 cm^{-1}, leading to water loss, Figure S1e. To construct the IRMPD spectrum in Figure 1b, the whole process is repeated, changing the irradiation wavenumber in Figure S1e. A series of mass spectra like the

one in Figure 1e, measured with the irradiation wavenumber changed sequentially, is obtained to generate IRMPD spectra.

In addition to using BIRD to dissociate the $Mn^+(H_2O)_8$ complex, IR radiation from the OPO laser system was also employed. The IRMPD spectrum of $Mn^+(H_2O)_8$, Figure 1c, shows a strong broad band at *ca.* 3200 cm^{-1}. This band was used to photodissociate the $Mn^+(H_2O)_8$ complex, forming $[Mn,(H_2O)_4]^+$. The IRMPD spectrum shown in Figure 1e was generated as follows: the mass-selected $Mn^+(H_2O)_8$ complex was irradiated for 3.0 s with IR light at 3200 cm^{-1}, leading to loss of 4 H_2O. The resulting $[Mn,(H_2O)_4]^+$ complex was mass-selected and subject to IR radiation for 0.3 s, generating the IRMPD spectrum in Figure 1e.

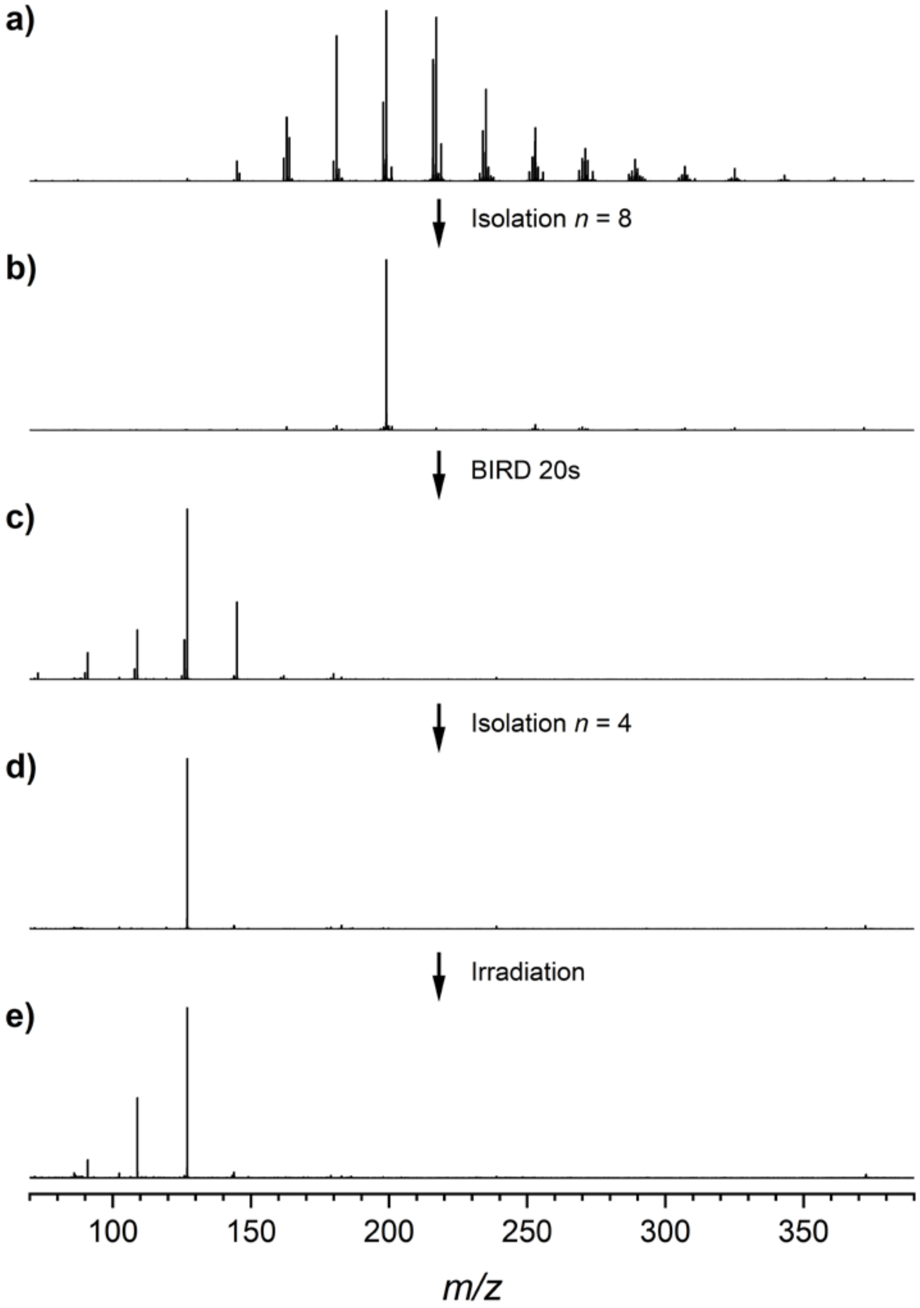

Figure S1: Representative mass spectra outlining the experimental procedure used to construct BIRD+IRMPD spectra. (a) mass spectrum obtained from the ion source, (b) mass-selected $Mn^+(H_2O)_8$ cluster, (c) mass distribution after 20 s irradiation with black-body infrared radiation, (d) mass-selected $Mn^+(H_2O)_4$ cluster, and (e) mass spectrum recorded after irradiation with infrared light at 3680 cm^{-1} for 0.3 s. Arrows indicate that mass spectra, a–e, were measured sequentially.

Table S1: Irradiation times and temperatures used in the experiment.

Cluster	OH Region	Temperature / K	Irradiation time / s
$Mn^{+}(H_2O)_4$	Stretch	87	0.2
		298	0.2
	Bend		0.3
$Mn^{+}(H_2O)_8$	Stretch	87	0.2
		298	0.2
	Bend		1.0
$Mn^{+}(H_2O)_4 \leftarrow Mn^{+}(H_2O)_8$	Stretch	87	3.0 @3200 cm^{-1}/0.3
		298	20.0/0.3
	Bend		20.0/10.0

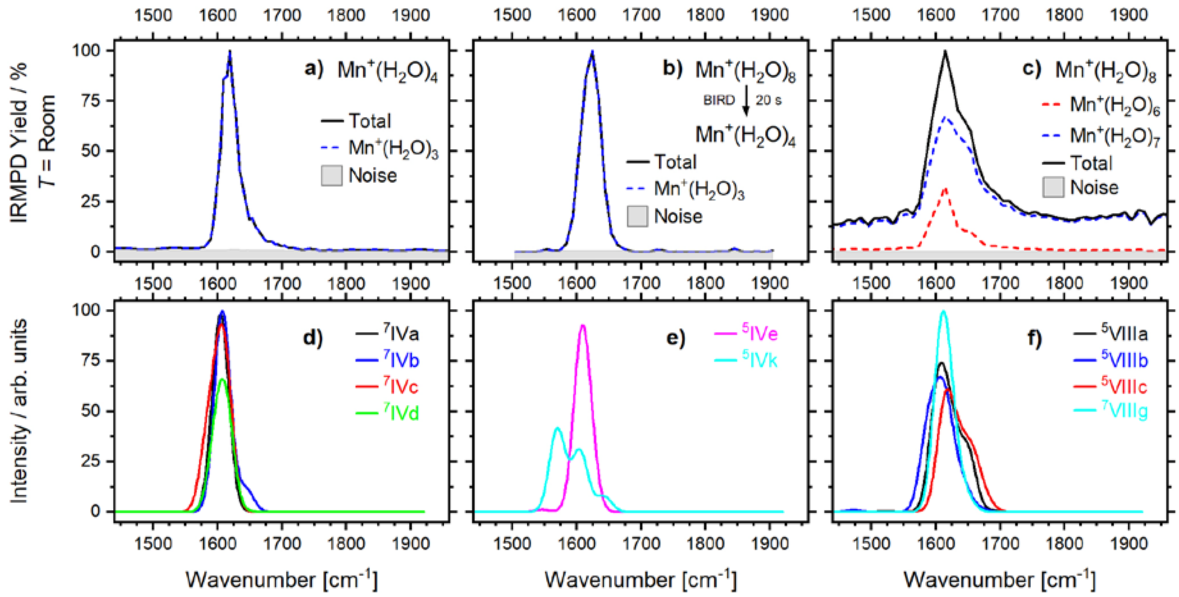

Figure S2. a) Experimental IRMPD spectrum of $Mn^{+}(H_2O)_4$ recorded at ≈ 298 K. b) Experimental IRMPD spectrum of $Mn^{+}(H_2O)_4$ recorded, and mass-selected, after $Mn^{+}(H_2O)_8$ was subject to 20.0 s of black-body-infrared-radiative dissociation (BIRD), at ≈ 298 K. c) Experimental IRMPD spectrum of $Mn^{+}(H_2O)_8$ recorded at ≈ 298 K. In d–f), simulated infrared spectra were modelled at the BHandHLYP/aug-cc-pVDZ level with a scaling factor of 0.96.

Computational Data

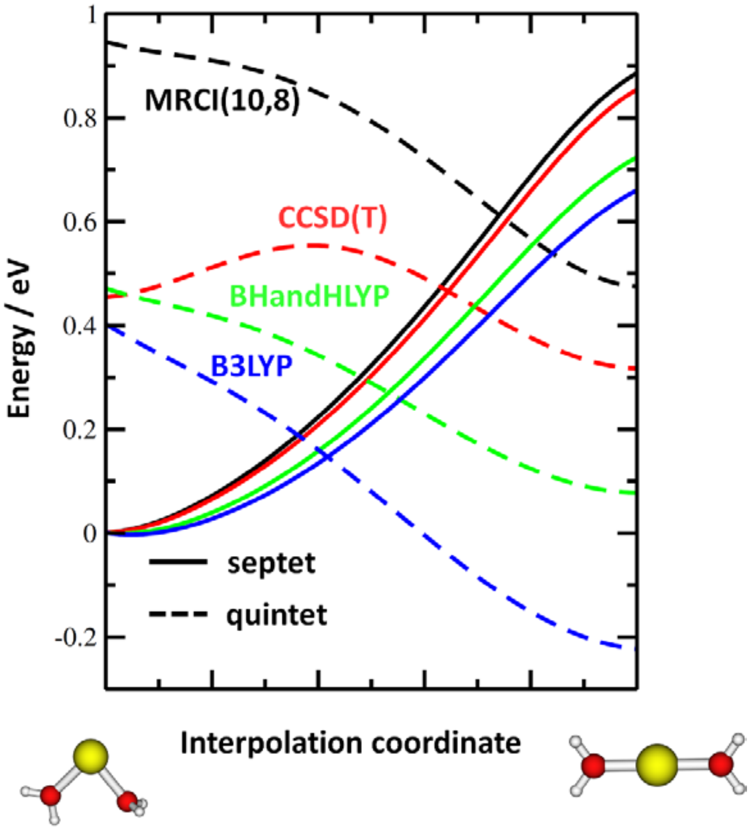

Figure S3. Interpolation between septet and quintet $Mn^+(H_2O)_2$ minima optimized at the CCSD/aug-cc-pVDZ level at different computational levels using the aug-cc-pVDZ basis set. State average of one quintet and one septet state was used for multi-reference calculations.

Table S2 - Relative stability of $Mn^+(H_2O)_2$ in septet spin multiplicity compared to the quintet analogue (in kJ mol^{-1}), negative values indicate that the ion in septet spin multiplicity is more stable. The structures were optimized in both spin multiplicities at the respective level using the aug-cc-pVDZ basis set, energies include zero-point correction. For the CCSD(T) value, the CCSD zero-point correction was used.

Method	ΔE
B3P86	4.7
B3LYP	21.6
BHandHLYP	-7.1
BMK	14.3
CAM-B3LYP	27.1
M06	57.3
M06L	33.3
M11	-0.9
MN15	32.2
O3LYP	15.3
*ω*B97XD	29.2
CCSD	-35.1
CCSD(T)	-28.7

Table S3 – Relative energies of $Mn^+(H_2O)_2$ and $HMnOH^+(H_2O)$ isomers as optimized at different levels of theory using the aug-cc-pVDZ basis set (in kJ mol^{-1}). See Figure S4 for the respective isomers.

Isomer	CCSD	BHandHLYP	B3LYP	M11
**^{7}IIa**	0.0	0.0	0.0	0.0
**^{7}IIb**	25.1	22.5	19.3	23.3
**^{5}IIc**	35.1	7.1	-21.6	0.9
**^{5}IId**	81.4	61.7	52.8	74.1
**^{5}IIe**	134.7	120.7	41.7	82.2

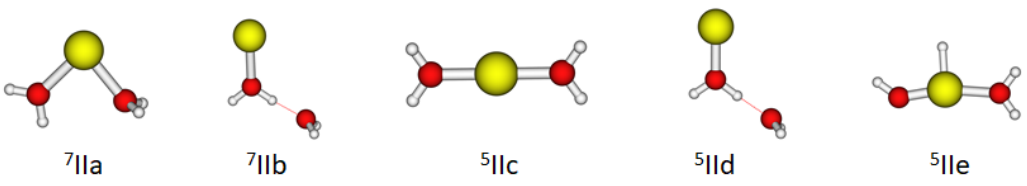

Figure S4. Selected low-energy structures of $Mn^+(H_2O)_2$ and $HMnOH^+(H_2O)$.

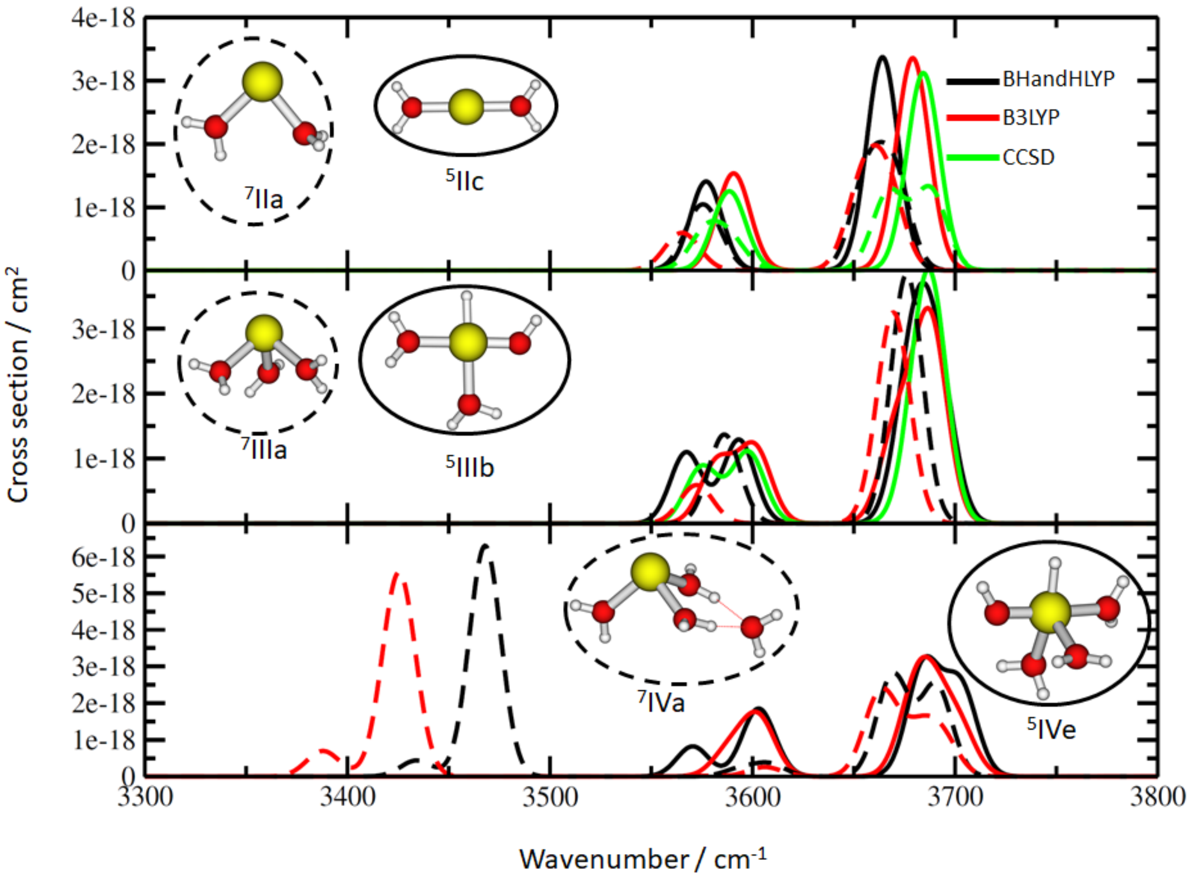

Figure S5. IR spectra of selected $Mn^+(H_2O)_n$ clusters, n = 2–4, in the O–H stretching region using various methods along with the aug-cc-pVDZ basis set. Scaling factors of 0.92, 0.96, and 0.95 were used for BHandHLYP, B3LYP, and CCSD, respectively.

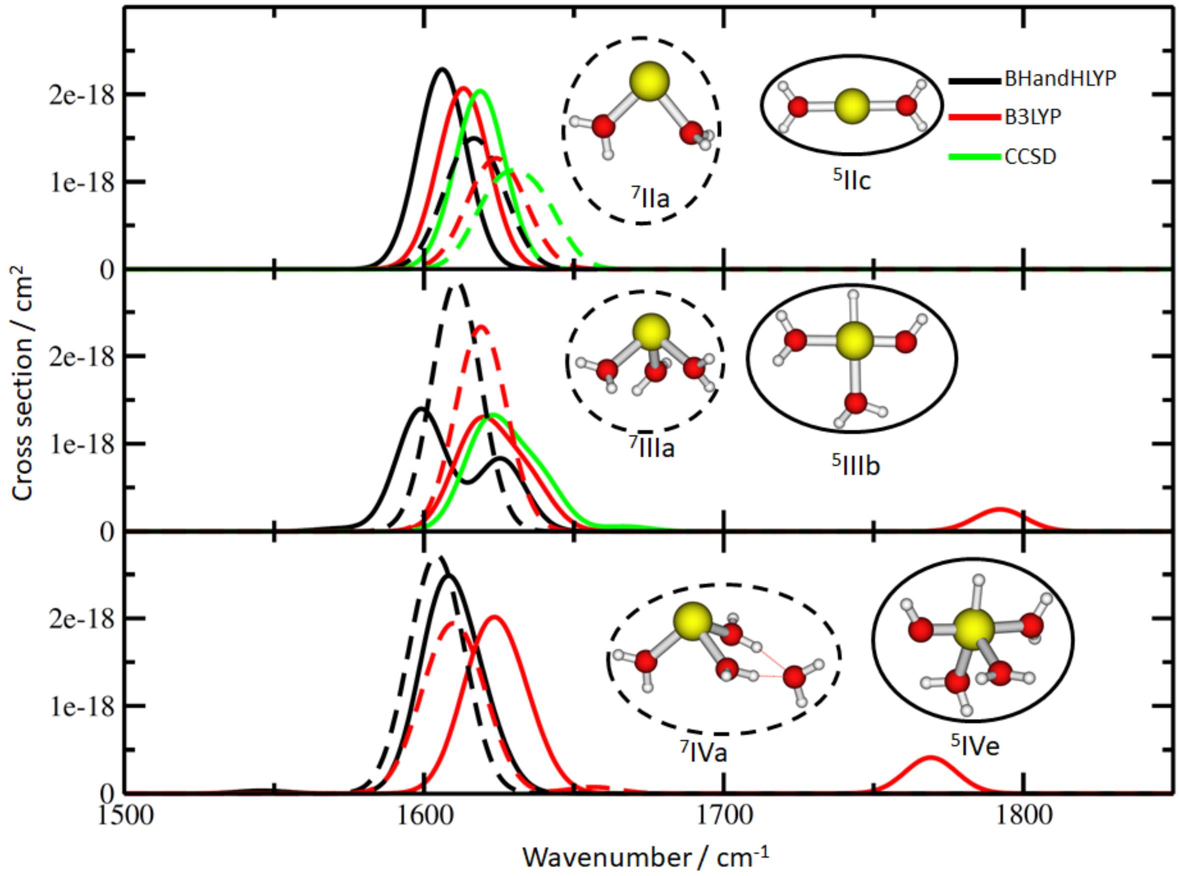

Figure S6. IR spectra of selected $Mn^+(H_2O)_n$ clusters, n = 2–4, in the H_2O scissoring region using various methods along with the aug-cc-pVDZ basis set. Scaling factors of 0.96, 1.00 and 0.98 were used for BHandHLYP, B3LYP and CCSD, respectively.

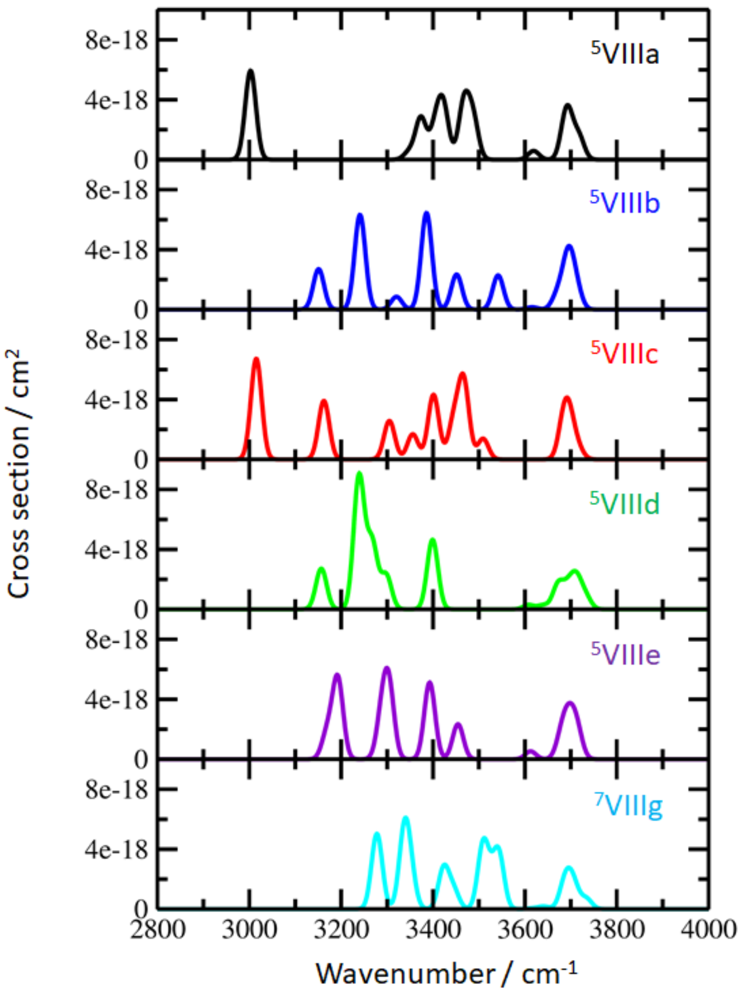

Figure S7. Calculated IR spectra of six selected $Mn^{+}(H_2O)_8$ isomers at the BHandHLYP/aug-cc-pVDZ level of theory.

Table S4 – Position (in cm^{-1}) and intensity (in km mol^{-1}, in parenthesis) of the Mn–H vibration for the most stable $HMnOH^{+}(H_2O)_n$, n = 1–3, isomers. The frequencies are unscaled. The aug-cc-pVDZ basis set was used.

method	^{5}Ic	^{5}IIe	^{5}III	^{5}IVe
CCSD	1466 (49.0)	1598 (17.4)	1701 (7.1)	–
BHandHLYP	1444 (127.9)	1519 (40.6)	1638 (4.2)	1610 (4.2)
B3LYP	1722 (6.5)	1759 (3.5)	1792 (32.2)	1769 (52.7)
B3P86	1768 (3.9)	1798 (4.6)	1828 (39.0)	1808 (59.9)
BMK	1662 (16.7)	1751 (4.1)	1827 (36.2)	1806 (65.2)
CAM-B3LYP	1704 (14.7)	1763 (3.0)	1812 (24.8)	1790 (43.1)
M06	1696 (13.9)	1754 (4.7)	1777 (12.0)	1754 (25.5)
M06L	1773 (4.3)	1780 (5.0)	1811 (36.0)	1791 (55.4)
M11	1610 (21.0)	1690 (5.1)	1767 (16.9)	1742 (29.4)
MN15	1723 (3.2)	1777 (3.5)	1830 (32.8)	1797 (49.1)
O3LYP	1698 (5.0)	1723 (2.2)	1742 (26.2)	1717 (41.5)
ωB97XD	1764 (4.3)	1808 (6.3)	1829 (39.4)	1807 (62.9)

Table S5 – Mn-H bond length (in Å) for the most stable $HMnOH^+(H_2O)_n$, n = 0–3, isomers. The aug-cc-pVDZ basis set was used.

method	^{5}Ic	^{5}IIe	^{5}III	^{5}Ive
CCSD	1.639	1.609	1.599	1.601
BHandHLYP	1.643	1.618	1.604	1.608
B3LYP	1.578	1.574	1.577	1.581
B3P86	1.564	1.562	1.568	1.571
BMK	1.589	1.580	1.581	1.586
CAM-B3LYP	1.575	1.568	1.572	1.575
M06	1.597	1.590	1.596	1.599
M06L	1.584	1.582	1.589	1.593
M11	1.585	1.572	1.573	1.577
MN15	1.564	1.560	1.563	1.569
O3LYP	1.580	1.577	1.581	1.585
ωB97XD	1.569	1.566	1.572	1.576

Table S6 – Position (in cm^{-1}) and intensity (in km mol^{-1}, in parenthesis) of several vibrations in $HMnOH^+(H_2O)$. The frequencies are unscaled. The aug-cc-pVDZ basis set was used.

Method	Mn-H	H_2O scissoring	OH stretch		
CCSD	1598 (17.4)	1674 (109.6)	3769 (189.5)	3858 (238.4)	3876 (273.7)
BHandHLYP	1519 (40.6)	1696 (125.9)	3876 (227.0)	3959 (274.0)	4001 (312.5)
B3LYP	1759 (3.5)	1637 (107.5)	3726 (216.0)	3811 (279.1)	3812 (263.9)
B3P86	1798 (4.6)	1637 (108.2)	3757 (221.0)	3843 (313.5)	3845 (235.4)
BMK	1751 (4.1)	1653 (124.8)	3815 (245.1)	3898 (288.6)	3938 (341.0)
CAM-B3LYP	1763 (3.0)	1637 (120.3)	3752 (230.6)	3833 (272.7)	3860 (320.1)

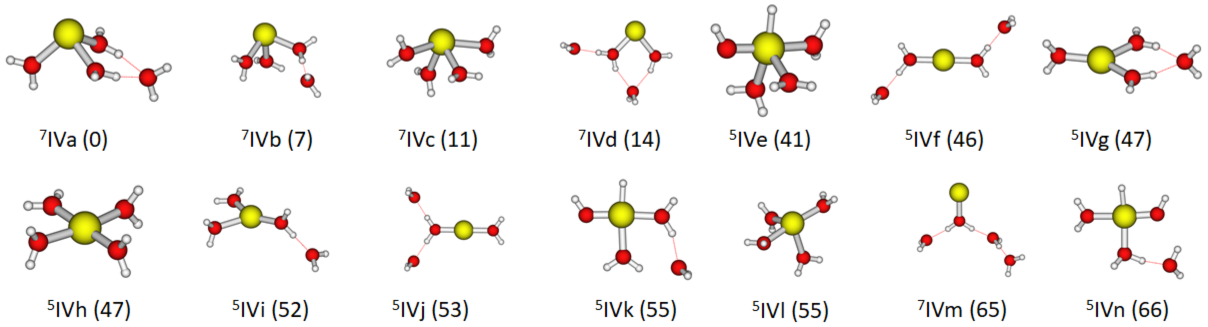

Figure S8. Selected low-energy structures of $Mn^+(H_2O)_4$ and $HMnOH^+(H_2O)_3$. Relative energies in kJ mol^{-1} were evaluated at the CCSD(T)/aug-cc-pVDZ//BHandHLYP/aug-cc-pVDZ level of theory.

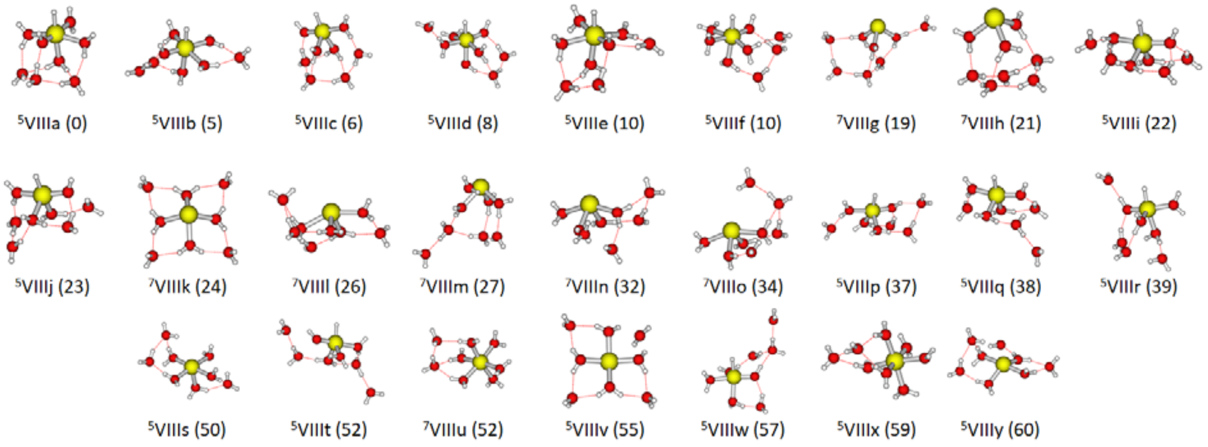

Figure S9. Selected low-energy structures of $Mn^+(H_2O)_8$ and $HMnOH^+(H_2O)_7$. Relative energies in kJ mol^{-1} were evaluated at the CCSD(T)/aug-cc-pVDZ//BHandHLYP/aug-cc-pVDZ level of theory.

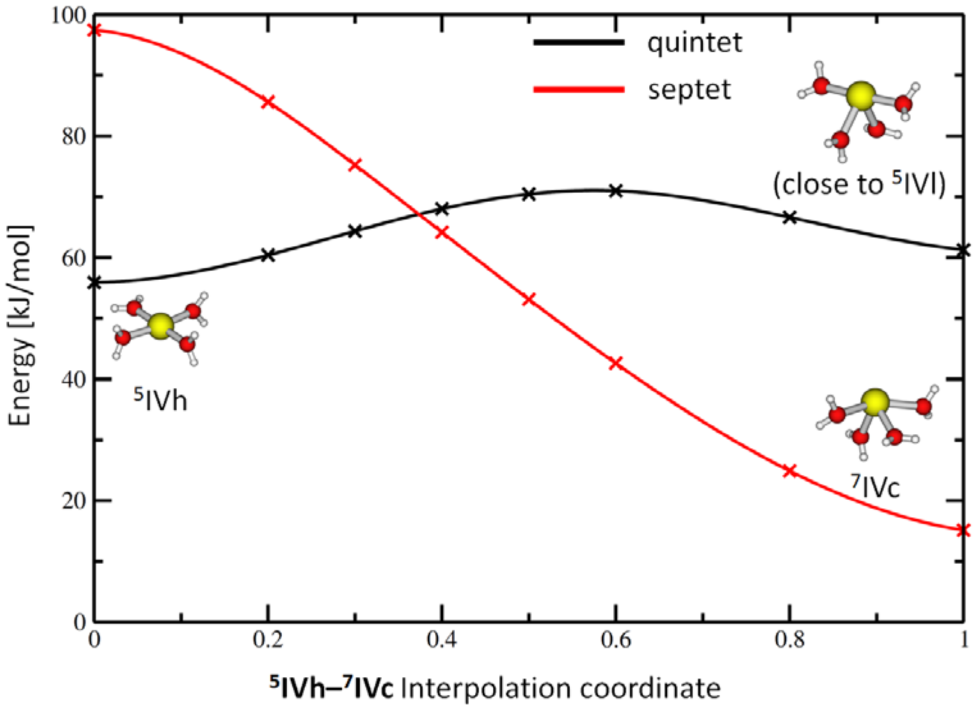

Figure S10. Interpolation between ^{5}IVh and ^{7}IVc minima optimized at the BHandHLYP/aug-cc-pVDZ level and single-point recalculated at the CCSD(T)/aug-cc-pVDZ level. Calculated points are shown as crosses, splines are included to guide the eye.

Calculated Structures and IR Spectra

Cartesian coordinates (in Å) of structures optimized at the BHandHLYP/aug-cc-pVDZ level along with zero-point corrected energies (in a.u.)

```
7IIa
E = -1303.560921
Mn 0.000000 0.746126 0.000000
O 1.523974 -0.827323 0.000000
O -1.500634 -0.905893 0.000000
H -2.016449 -1.161965 0.766977
H -2.016449 -1.161965 -0.766977
H 1.373818 -1.773738 0.000000
H 2.472361 -0.689762 0.000000

7IIb
E = -1303.552354
O 0.000000 0.956251 0.000000
Mn -1.106651 -0.837562 0.000000
O 2.593518 1.010962 0.000000
H 3.141336 1.166519 0.767036
H 3.141336 1.166519 -0.767036
H 0.988143 1.022193 0.000000
H -0.352673 1.846108 0.000000

5IIc
E = -1303.558205
Mn 0.000000 0.000000 0.000000
O -0.000000 -0.000000 2.047336
O 0.000000 -0.000000 -2.047336
H 0.000000 0.775759 -2.610115
H -0.000000 -0.775759 -2.610115
H -0.000000 -0.775759 2.610115
H -0.000000 0.775759 2.610115

5IId
E = -1303.537409
O -0.000000 0.901170 0.000000
Mn -1.099164 -0.799241 0.000000
O 2.574068 0.973785 0.000000
H 3.124085 1.116201 0.768064
H 3.124085 1.116201 -0.768064
H 0.992346 0.958574 0.000000
H -0.353957 1.790418 0.000000

5IIe
E = -1303.514934
Mn 0.000000 0.123483 0.000000
O -1.040801 1.523559 0.000000
O 0.737211 -1.766036 0.000000
H 1.676705 -1.963729 0.000000
H 0.262150 -2.600184 0.000000
H -0.902247 2.468011 0.000000
H 1.392115 0.948657 0.000000

7IIIa
E = -1379.969596
Mn -0.000469 0.000600 -0.780216
O 1.336040 1.134561 0.608760
H 0.999193 1.734173 1.275561
O 0.316136 -1.724006 0.607903
O -1.651164 0.587995 0.609450
H 2.201405 1.458391 0.356820
H -2.364556 1.175723 0.358780
H -2.001454 -0.004714 1.275487
H 0.163596 -2.635524 0.356803
H 1.005447 -1.731437 1.273037

5IIIb
E = -1379.940160
Mn -0.008482 -0.365981 -0.000002
O -1.775979 -0.563327 -0.000087
H -2.241391 -1.395456 0.000250
O -0.431783 1.707654 0.000081
O 2.017654 -0.109271 -0.000012
H 0.236621 -1.951062 0.000093
H 2.613825 -0.859487 0.000389
H 2.544109 0.690186 -0.000445
H -0.032782 2.575950 -0.000195
H -1.387460 1.808934 0.000086

7IVa
E = -1456.371756
O -1.993825 -0.005746 1.255313
Mn -0.940386 0.003137 -0.702112
O 0.574161 -1.437927 0.030908
O 0.575078 1.438510 0.040033
H -1.626446 -0.011444 2.138876
H -2.946687 -0.004925 1.340398
H 1.499987 1.185000 0.153204
H 0.538054 2.384291 -0.090373
H 0.536959 -2.382588 -0.107339
H 1.499334 -1.185180 0.143931
O 2.961489 -0.002155 0.323393
H 3.450814 -0.005820 1.145500
H 3.622413 0.000789 -0.368567

7IVb
E = -1456.369339
O -1.477640 -0.794265 1.366107
Mn -1.029356 0.008600 -0.677134
O -0.215311 1.793396 0.371788
O 0.867980 -1.047527 -0.435071
O 3.206628 0.031840 0.283348
H -2.357691 -0.867572 1.735091
H -0.963845 -1.521319 1.718368
H -0.137918 1.886640 1.321190
H -0.203949 2.673864 -0.002155
H 1.722989 -0.645671 -0.180534
H 3.786911 -0.346526 0.940490
H 3.763444 0.537757 -0.304678
```

H 1.070690 -1.799728 -0.988798

7IVc
E = -1456.366345
O 0.004253 1.732277 0.758682
Mn -0.000589 0.009299 -0.672864
O -2.306575 -0.263760 -0.142287
O -0.001439 -1.268349 1.142897
H 0.003616 1.670108 1.713740
H 0.003613 2.660712 0.528877
H -0.776301 -1.766333 1.399978
H 0.773768 -1.765573 1.400408
H -2.854550 0.406201 0.267178
H -2.838712 -0.647485 -0.839913
O 2.305343 -0.267492 -0.143149
H 2.836656 -0.657036 -0.838166
H 2.853965 0.405541 0.260350

7IVd
E = -1456.367513
O 2.098955 0.071238 0.000471
Mn 0.493322 -1.397725 -0.000067
O -0.774430 0.306309 -0.000604
O 0.957302 2.579151 -0.000082
H 3.041461 -0.084366 0.000502
H -0.452078 1.212677 -0.000840
H -1.752105 0.309248 -0.000241
H 1.016107 3.154626 -0.762040
H 1.014926 3.154762 0.761862
H 1.934939 1.025599 0.000595
O -3.425717 0.262794 0.000346
H -3.992563 0.207763 0.766140
H -3.992605 0.206887 -0.765355

5IVe
E = -1456.352768
O -1.828309 -0.080628 -0.597301
Mn -0.034261 0.008173 -0.415668
O 0.015387 1.703431 0.974463
O -0.293555 -1.483983 1.124363
O 2.026372 -0.266002 -0.347202
H -2.297234 0.086253 -1.409238
H 2.472300 -0.968852 0.125065
H 2.576505 -0.021948 -1.091517
H -0.044973 -1.543588 2.045239
H -1.222712 -1.720090 1.060010
H -0.824898 2.144632 1.100408
H 0.702232 2.306001 1.253812
H 0.136154 0.530728 -1.926669

5IVf
E = -1456.364577
O 1.821515 -0.850601 -0.000237
Mn 0.000032 -0.000091 -0.000357
O -1.821477 0.850301 -0.000519
O -4.141589 -0.356903 0.000848
H 2.693478 -0.394536 0.000036
H -2.693528 0.394325 0.000008
H -1.972213 1.793881 -0.000864
H -4.674448 -0.551166 0.768564
H -4.674844 -0.552194 -0.766332
H 1.972422 -1.794150 -0.001791
O 4.141401 0.357524 0.000765
H 4.675314 0.552035 -0.766162
H 4.674212 0.551512 0.768595

5IVg
E = -1456.362308
Mn 0.826976 -0.270552 0.001334
O -0.952326 -1.400432 -0.001756
O 2.817113 0.359330 -0.001108
O -0.593154 1.416628 -0.000887
H -0.409225 2.353266 -0.002285
H -1.550004 1.294453 -0.000156
H 3.375379 0.277640 -0.776891
H 3.377325 0.282054 0.773724
H -1.018252 -2.352287 -0.001411
H -1.841090 -1.019772 -0.001135
O -3.163356 0.279641 0.000460
H -3.737502 0.343732 -0.762152
H -3.737243 0.343378 0.763293

5IVh
E = -1456.361415
Mn 0.000759 0.063342 -0.000189
O 0.018703 2.235667 -0.000068
O -0.018571 -2.111039 -0.000028
O 2.198044 -0.192867 0.000307
O -2.200768 -0.156110 0.000426
H -2.743419 0.013045 0.771715
H -2.742947 0.013553 -0.771089
H 0.024367 2.798414 -0.775282
H 0.024635 2.799601 0.774273
H 2.742806 -0.035013 0.772500
H 2.744464 -0.033335 -0.770360
H 0.749047 -2.680069 -0.001471
H -0.797179 -2.664956 -0.000660

5IVi
E = -1456.361189
O 2.189132 -1.240563 -0.369315
Mn 0.459655 -0.158682 0.204120
O -1.468230 0.082846 0.813652
O 1.894902 1.521885 -0.291766
H 3.010064 -0.798498 -0.586284
H 2.343406 -2.183337 -0.373206
H 1.809341 2.053385 -1.084327
H 2.240328 2.100553 0.389259
H -1.720581 0.046999 1.735131
H -2.283380 0.040918 0.268831
O -3.678825 -0.015859 -0.619111
H -4.144259 -0.804203 -0.888797
H -4.242115 0.724748 -0.831290

5IVj
E = -1456.361543
O 3.126926 0.000242 -0.000050
Mn 1.074686 0.000143 0.000017
O -0.905073 0.000005 0.000173
O -2.375082 -2.214299 -0.000493

H 3.687307 0.777039 -0.008496
H -1.468462 -0.797912 -0.000303
H -1.468803 0.797654 0.000064
H -2.765243 -2.627954 0.765859
H -2.768110 -2.628323 -0.765173
H 3.687391 -0.776497 0.008044
O -2.376190 2.213740 0.000195
H -2.768037 2.627922 -0.765012
H -2.767842 2.627001 0.766001

5IVk
E = -1456.348268
O 0.885378 -1.355818 -0.000201
Mn -0.858521 -0.336280 -0.000353
O 0.353926 1.378818 0.001662
O -2.314843 0.679144 -0.001201
O 2.983783 0.350662 -0.000610
H -0.065565 2.238991 0.001588
H 1.311244 1.463050 -0.000095
H -3.211571 0.356555 0.000537
H 0.977067 -2.306526 0.002188
H 1.757872 -0.924246 0.000539
H 3.557950 0.431668 0.761336
H 3.557823 0.429392 -0.762889
H -1.687745 -1.704330 0.008438

5IVl
E = -1456.356783
O -0.004780 1.922148 -0.142752
Mn 0.001958 -0.287186 0.458927
O 2.148192 -0.381031 0.414769
O -0.003291 -0.489203 -1.830240
H -0.005774 2.214208 -1.054848
H -0.004522 2.704132 0.408658
H 0.761086 -0.824603 -2.298901
H -0.771881 -0.821763 -2.294021
H 2.724232 0.381087 0.496484
H 2.539190 -1.086561 0.933692
O -2.144609 -0.385739 0.419946
H -2.533515 -1.091665 0.939853
H -2.721870 0.375404 0.502121

7IVm
E = -1456.349897
O 0.522737 0.364900 -0.181555
Mn 1.730653 -1.255305 0.138920
O 1.377195 2.856629 0.196198
O -1.882859 -0.065755 -0.983391
H -0.417773 0.288415 -0.486300
H 0.764085 1.303709 -0.061314
H 1.663497 3.452590 -0.492418
H 1.203977 3.395512 0.964846
H -2.671365 -0.104415 -0.419284
H -2.184567 0.069468 -1.877327
O -4.069768 -0.220616 0.622957
H -4.609879 0.505937 0.923923
H -4.592730 -1.009865 0.741210

5IVn
E = -1456.344540
O -0.446723 -1.354652 -0.086293
Mn 0.629924 0.410330 -0.036029
O -0.835000 1.452235 0.011852
O 2.344980 -0.691235 0.109945
O -2.831865 -0.512710 0.033810
H -0.775559 2.403845 0.001623
H 3.222110 -0.308064 0.086740
H 2.418113 -1.602605 0.393993
H -0.328584 -2.151313 -0.599183
H -1.428754 -1.180947 -0.000238
H -3.626178 -0.702379 0.526272
H -2.651163 0.429012 0.089834
H 1.570779 1.705089 -0.152833

TS1
E = -1456.363170
o 2.677246 1.145571 0.000111
mn 0.688450 -0.937897 -0.000058
o 0.002349 1.093801 -0.000122
o -1.497862 -1.277720 0.000125
o -2.786430 1.213958 0.000011
h 3.252394 1.230908 -0.759059
h 3.252265 1.230964 0.759374
h 0.679011 1.771972 -0.000068
h -0.886202 1.464821 -0.000091
h -3.282264 1.512824 0.761629
h -3.282322 1.512723 -0.761608
h -2.150861 -0.565600 0.000107
h -1.955712 -2.116076 0.000177

TS2
E = -1456.369392
o 3.105931 -0.030471 0.289145
h 0.338340 1.779355 1.261443
h 3.696188 -0.500380 0.874968
h 3.661074 0.524232 -0.255411
h 1.661126 -0.710801 -0.301710
o 0.796602 -1.067668 -0.586409
h 0.969759 -1.757476 -1.224592
mn -1.066555 0.073509 -0.629485
o -1.394537 -0.845623 1.383903
h -0.937601 -1.649447 1.631722
h -2.230840 -0.842756 1.849108
o -0.051045 1.773213 0.387111
h -0.149777 2.683937 0.111599

TS3
E = -1456.364319
o 2.473049 0.086495 0.069146
mn -0.220274 -0.004305 -0.735543
o -0.157473 1.722133 0.676841
o 0.418670 -1.540256 0.738271
o -2.240639 -0.271814 0.303029
h -2.394943 -0.860745 1.041908
h -3.067755 -0.203163 -0.173765
h -0.943790 1.944325 1.175310
h 0.298377 2.542385 0.492338
h 0.281588 -2.484908 0.684539
h 1.362936 -1.387732 0.846654
h 2.935309 0.671912 0.667689

h 3.086277 -0.086921 -0.644389

TS4
E = -1456.334467
o 1.766293 0.796362 -0.734738
h 0.996278 0.248427 -1.708120
mn 0.012199 0.038910 -0.196490
o -1.253468 1.512028 0.820281
o -1.717883 -1.188899 -0.564321
o 1.268389 -1.200716 1.073898
h 1.253518 -2.009525 1.580793
h 2.183451 -0.967411 0.904953
h 1.998940 1.619703 -1.158643
h -0.910088 2.233226 1.346994
h -2.156061 1.736460 0.597258
h -2.335453 -1.620571 0.024399
h -1.842212 -1.563250 -1.436349

TS5
E = -1456.343124
o 2.434380 -0.330075 -1.001753
mn -0.464492 -0.300501 0.311362
o -0.830038 1.640363 -0.446734
o 1.234874 0.367744 1.196858
o -1.919565 -0.773641 -0.605275
h -2.321149 -1.637267 -0.582609
h -0.366297 -1.731399 1.023672
h 1.506104 0.155994 2.089254
h 1.974442 0.192421 0.582876
h 2.861734 -1.171179 -1.158671
h 2.777083 0.263206 -1.668676
h -0.541236 2.529127 -0.248528
h -1.635582 1.676500 -0.966131

5VIIIa
E = -1761.958550
O 0.116728 0.008489 -1.346477
Mn -1.016774 0.002484 0.451487
O -2.687122 0.019725 -0.798270
O -0.851678 -2.245652 0.060115
O 0.398268 0.003009 1.688239
O -0.872126 2.232525 0.044163
O 2.784167 -0.009964 0.669981
O 1.778546 -2.084261 -0.842136
H 0.213692 0.032626 2.620561
H -2.065138 -0.036423 1.671677
H 0.044439 2.511431 -0.068484
H -1.324482 2.910107 0.539516
H -3.570789 -0.272755 -0.588771
H -2.543673 -0.079691 -1.738484
H 0.678590 0.790597 -1.451217
H 0.696741 -0.758905 -1.458890
H 0.069268 -2.479116 -0.115438
H -1.184648 -2.881546 0.688622
H 2.360338 -1.528526 -0.299809
H 2.328532 -2.693380 -1.327873
H 1.956417 -0.005504 1.211205
H 3.522015 -0.015964 1.274112
O 1.777028 2.100394 -0.810111
H 2.327655 2.714468 -1.288871
H 2.359896 1.536381 -0.279049

5VIIIb
E = -1761.957808
O -1.758347 1.268356 -0.370410
Mn -0.259501 -0.182760 -0.598519
O 0.963634 -1.582719 -0.857464
O 0.932728 1.693406 -1.026263
O -1.806244 -1.483413 0.411979
O 2.824817 1.370315 0.869023
O -4.077073 0.207973 0.733043
O 3.250585 -1.172551 0.474175
H 0.981035 -2.044653 -1.688759
H -1.524083 2.191834 -0.324375
H -2.619274 1.118405 0.038147
H 1.734752 1.765714 -0.482353
H 1.181175 1.893464 -1.925722
H -1.779934 -2.427858 0.278203
H -2.713432 -1.221376 0.587663
H 3.968859 -1.778512 0.323153
H 2.494016 -1.454194 -0.076762
H 3.238777 0.493384 0.724799
H 3.452459 1.932213 1.314702
H -4.857324 0.157131 0.182489
H -4.393934 0.416656 1.610682
H -0.723563 -0.380414 -2.131502
O 0.447552 0.180070 1.405637
H 0.538169 -0.604671 1.943198
H 1.288610 0.660382 1.471668

5VIIIc
E = -1761.959167
Mn 0.098066 -1.434137 0.028562
O -3.159412 0.695858 -0.024923
O -0.224646 0.034363 1.637224
O 1.201167 2.283242 1.047267
O 2.747878 0.868904 -0.695732
O -1.069655 2.521029 -0.604716
O 0.176908 0.171240 -1.330385
O -1.895845 -1.560677 -0.242632
O 1.944516 -1.532963 -0.025598
H -4.035612 0.928160 -0.320009
H -2.596715 1.469309 -0.137842
H 1.084510 0.480323 -1.459992
H -0.375340 0.963429 -1.271055
H 1.909598 1.924301 0.484921
H 1.609810 2.896434 1.653698
H -2.413345 -2.336349 -0.045361
H -2.460433 -0.751505 -0.179565
H -0.362318 2.713312 0.024238
H -1.199662 3.309715 -1.126578
H 0.312728 0.842919 1.634568
H -0.441921 -0.178420 2.541146
H 2.399727 -2.319734 0.254276
H 2.728831 -0.067801 -0.405898
H 3.582130 1.028581 -1.128695
H 0.039066 -2.897218 0.684058

5VIIId
E = -1761.957963

Mn -0.358247 -0.333416 -0.308974
O 0.700602 0.110158 1.497030
O 0.228763 1.816320 -0.701888
O 1.033881 -1.210016 -1.212982
O -0.499034 -2.360615 0.814361
O -2.030293 0.314976 0.680504
H 0.918318 -1.441192 -2.128710
H -1.295944 -0.533846 -1.600155
H 0.046047 -2.940986 0.283156
H -1.217987 -2.878225 1.169090
H -1.899790 0.917047 1.410107
H -2.908677 0.485252 0.289575
H 0.976956 -0.649709 2.005540
H 1.449766 0.729041 1.438495
H 1.107940 2.053575 -0.369539
H 0.133801 2.211027 -1.564788
O 2.591284 1.804658 0.646476
H 3.142805 1.109321 0.229914
H 3.167722 2.455870 1.036330
O 3.473606 -0.413149 -0.400746
H 2.671920 -0.797875 -0.801916
H 4.231925 -0.758136 -0.860937
O -4.451233 0.772891 -0.297147
H -4.701286 1.358498 -1.007459
H -5.247941 0.333953 -0.009209

5VIIIe
E = -1761.956073
O -0.493145 -0.443183 1.467761
Mn 0.494441 -0.586718 -0.414611
O 0.932284 -2.601474 0.113728
O -1.552627 -1.399056 -0.954063
O 0.266497 1.200481 -1.053201
O 2.243191 -0.113487 0.839527
O 2.481332 2.374428 0.142471
O -2.025178 2.321964 -0.098068
O -3.016291 0.082767 0.798408
H 0.396865 1.332331 -1.988146
H 1.158450 -0.952514 -1.840740
H 2.553122 0.808472 0.724971
H 2.991863 -0.670000 1.034589
H 1.188146 -3.260857 -0.528424
H 0.319612 -3.014518 0.721678
H -0.041699 0.005828 2.179135
H -1.441397 -0.222701 1.494946
H -2.286864 -0.980317 -0.478686
H -1.835752 -1.531798 -1.855264
H -2.887523 1.001740 0.483583
H -3.870403 0.022348 1.217482
H -1.196726 2.046136 -0.527668
H -2.305456 3.144472 -0.486826
H 1.638646 2.301539 -0.323296
H 2.569594 3.258285 0.485434

5VIIIf
E = -1761.954790
Mn 0.899172 -0.403129 -0.183938
O 0.797907 1.501366 0.781905
O 2.689898 0.653610 -1.184676
O -0.113384 0.012808 -1.719592
O -0.686306 -0.915019 1.243817
O 2.301063 -0.926086 1.316939
H 2.420483 -1.846296 1.547142
H 2.235695 -0.435158 2.135788
H -1.425467 -1.409211 0.839439
H -1.110513 -0.170784 1.678708
O -1.770140 1.735647 1.350528
H -2.301589 2.331573 1.871296
H -2.236386 1.566258 0.512674
H -0.093894 1.814780 1.029118
H 1.278729 2.221270 0.379608
O -2.560123 0.833659 -1.079382
H -1.672320 0.583982 -1.424343
H -3.005652 1.315177 -1.771240
H 2.497786 0.822113 -2.106162
H -0.185078 -0.647204 -2.402575
H 3.625146 0.472602 -1.117064
H 1.123734 -1.891455 -0.760074
H -3.071948 -0.998173 -0.552592
O -2.785148 -1.807065 -0.118417
H -3.548159 -2.362605 0.009767

7VIIIg
E = -1761.959040
O -4.766373 0.581283 -0.605728
Mn -0.999465 -1.333605 0.529355
O 0.119890 -0.059688 1.951071
O -2.101754 0.344965 -0.240029
O 0.491981 -0.786486 -0.974453
O 3.178952 -1.746005 -1.052557
O 1.562491 2.073331 0.953789
H 1.384103 -1.136013 -1.065324
H 0.459863 0.103627 -1.347309
H -1.649997 1.068956 -0.685127
H -3.060321 0.432160 -0.365610
H -5.274004 0.058311 -1.221282
H -5.387111 0.929312 0.029441
H 0.628906 0.733527 1.719329
H 0.053045 -0.114073 2.900783
H 3.526316 -1.996218 -1.907470
H 3.316175 -2.505726 -0.488262
H 2.421950 1.709861 0.672541
H 1.748445 2.848849 1.477629
O 3.776726 0.866476 0.009411
H 4.697345 1.105538 0.067186
H 3.735520 -0.032438 -0.326931
O -0.030358 1.897666 -1.306156
H 0.516217 2.214471 -0.574015
H 0.017739 2.547644 -2.002234

7VIIIh
E = -1761.957827
O 1.025916 -1.236926 -1.458104
Mn 2.332732 -0.248534 0.013011
O 0.743088 -0.576565 1.467344
O 1.473254 1.759822 -0.263984
O -1.639252 -1.863394 -1.089224
O -1.386983 1.055419 2.071708
O -1.203150 2.394363 -0.333867
H 2.033858 2.506249 -0.462813

H 0.547673 2.040375 -0.364780
H 1.357755 -1.591475 -2.279640
H 0.094789 -1.498708 -1.367935
H 0.133858 0.106497 1.785380
H 0.314295 -1.410436 1.659884
H -1.531482 1.574820 2.858990
H -1.457773 1.656790 1.315077
H -1.466975 3.300018 -0.479286
H -1.684238 1.855077 -0.993065
H -1.978798 -2.676175 -1.457072
H -1.849484 -1.879787 -0.139833
O -2.382177 0.697347 -2.006662
H -3.106976 0.784002 -2.618618
H -2.265048 -0.239541 -1.817471
O -1.981397 -1.656553 1.613089
H -2.578551 -2.167539 2.153867
H -2.075605 -0.734924 1.879633

5VIIIi
E = -1761.953598
O 1.903665 -0.557737 0.888075
Mn 0.095042 -0.124315 0.625381
O -1.847652 0.538491 0.643586
O 0.560802 1.585364 -0.465777
O -0.452195 -1.466556 -0.970526
O -1.931834 2.930760 -0.785212
O 3.084552 1.778636 0.000990
O -3.228314 -1.787294 0.106363
O 1.894218 -2.606783 -1.040932
H -2.070475 1.324363 0.127204
H -2.515904 -0.147539 0.503293
H 2.147272 -0.939612 1.726614
H -0.036021 2.284488 -0.733875
H 1.498230 1.876066 -0.461797
H -1.255488 -1.980076 -0.893925
H 0.293107 -2.051890 -1.208226
H -4.004760 -1.876455 -0.443702
H -3.373457 -2.374161 0.847258
H -2.140610 3.724717 -0.294703
H -2.314635 3.055820 -1.652206
H 3.922862 1.873249 -0.440912
H 3.031369 0.888823 0.373725
H 2.242913 -1.974403 -0.401436
H 2.604340 -2.884641 -1.611161
H -0.270746 -1.009923 1.916793

5VIIIj
E = -1761.952832
Mn -0.362854 0.254244 -0.992286
H -2.798344 0.279035 -1.353871
O 0.096007 1.014430 0.868055
O 1.521996 0.747864 -1.542502
O 0.222069 -1.810309 -0.360854
H 0.442859 -2.435890 -1.049092
H -0.578224 -2.161086 0.097640
O -2.065294 -2.163383 0.793902
H -2.475116 -1.402495 0.362037
H -2.733386 -2.821941 0.959121
O -2.194160 0.202892 -0.620905
H -0.682527 0.042758 -2.551717
H 0.962833 1.323689 1.143247
H -0.634019 1.413705 1.387804
O -2.202524 1.888935 1.634326
H -2.623075 1.370267 0.940982
H -2.787419 1.919150 2.385668
O 2.852301 1.123955 0.666990
H 3.003874 0.216053 0.989318
H 3.607433 1.656285 0.903657
H 2.189781 0.969714 -0.856326
H 1.884646 0.826611 -2.419970
H 2.983530 -2.069980 1.882643
O 2.702796 -1.469305 1.197949
H 1.842975 -1.762611 0.890313

7VIIIk
E = -1761.954013
O 1.995792 0.146957 -0.289395
Mn -0.000090 0.000023 0.734494
O -0.146924 1.996180 -0.289084
O -1.995863 -0.146804 -0.289158
O 0.146789 -1.996340 -0.288641
O 2.954046 -2.548547 -0.183145
O -2.953785 2.548870 -0.183109
O -2.549033 -2.953618 -0.182821
H -2.486386 -0.971448 -0.273074
H -2.601812 0.597180 -0.273361
H 2.601591 -0.597135 -0.273895
H 2.486330 0.971621 -0.273195
H -0.597141 -2.602336 -0.273175
H 0.971596 -2.486619 -0.273296
H -0.971592 2.486604 -0.273992
H 0.597024 2.602092 -0.273569
H -3.339920 2.882290 0.624967
H -3.438180 2.966292 -0.892956
H -2.966715 -3.437259 -0.893037
H -2.882593 -3.340343 0.624919
H 3.438271 -2.966358 -0.892882
H 3.340413 -2.881580 0.624984
O 2.549218 2.953242 -0.182609
H 2.882157 3.339869 0.625391
H 2.967297 3.437047 -0.892476

7VIIIl
E = -1761.953392
O 1.953395 -1.878646 0.334847
Mn 0.172176 -0.457513 0.689746
O 1.598463 0.976296 -0.011103
O -1.425038 1.234079 -0.082906
O -0.482597 -1.339186 -1.215837
O -3.114408 -0.794655 -1.444269
O 4.168497 -0.238426 -0.288480
O -3.506427 -0.109999 1.290290
H -2.194412 1.076555 0.477270
H -1.736487 1.037625 -0.968894
H 2.083709 -2.617176 0.926124
H 2.821640 -1.499804 0.150923
H -1.429628 -1.342170 -1.428984
H -0.083078 -2.136116 -1.555220
H 1.369601 1.911734 -0.156488
H 2.534156 0.830879 -0.161167

H -4.278572 0.127737 1.800838
H -2.943458 -0.620075 1.877409
H -3.527088 -0.750857 -0.575879
H -3.788906 -1.050431 -2.067947
H 4.597384 -0.302726 -1.140252
H 4.865185 -0.048841 0.337869
O 0.466592 3.341737 -0.393377
H 0.558542 4.241601 -0.093926
H -0.420787 3.050290 -0.178645

7VIIIm
E = -1761.954747
O -2.167641 -2.160891 0.956482
Mn -1.866996 -0.450763 -0.770700
O -2.189647 0.692937 1.111262
O 0.117170 -1.064391 -0.060484
O -0.680707 1.315047 -1.577604
O 0.024699 1.882106 1.996618
O 1.645837 2.147530 -0.307444
H 0.098808 1.761988 -1.218631
H -0.953034 1.768819 -2.371352
H -2.548465 -1.723942 1.718214
H -2.716235 -2.922328 0.774559
H -1.417957 1.160351 1.489723
H -2.930981 1.292221 1.162671
H 1.029171 -0.897557 -0.335195
H 0.098922 -1.803733 0.543192
H 2.184788 2.906203 -0.517523
H 2.187138 1.360746 -0.467551
H 0.685947 2.094999 1.330483
H 0.194034 2.423820 2.761443
O 2.703381 -0.359185 -0.826772
H 2.896981 -0.541020 -1.744055
H 3.447263 -0.730433 -0.322792
O 4.784399 -1.404726 0.568442
H 4.950302 -2.329920 0.731886
H 5.568279 -0.938553 0.848435

7VIIIn
E = -1761.950861
O 1.892990 0.389579 0.408386
Mn 0.124254 1.048986 -0.807319
O -0.445023 -1.138452 -0.534724
O -1.008728 1.276269 1.043396
O 1.272481 -2.196040 1.408753
O -3.221873 -0.233415 1.268810
O 3.456379 -1.854303 -0.448938
O -3.190588 -1.661153 -1.219863
H -1.310057 -1.482789 -0.773089
H 1.603056 -0.150681 1.149484
H 2.555100 -0.160140 -0.025031
H -1.838822 0.792705 1.205621
H -1.080836 2.150594 1.418425
H 1.182654 -2.771415 2.164066
H 2.061601 -2.469877 0.934953
H -3.581161 -2.525229 -1.337503
H -3.400678 -1.172748 -2.014674
H -3.438655 -0.719365 0.469053
H -4.033890 -0.095881 1.748085
H 4.391830 -1.940328 -0.273642
H 3.334333 -2.168273 -1.343465
H -0.076097 -1.674024 0.176697
O 0.956915 3.082961 0.141007
H 1.817935 2.964501 0.541536
H 1.006916 3.874726 -0.392164

7VIIIo
E = -1761.950529
O -1.958896 2.994714 -0.580825
Mn -0.710847 -0.590041 -0.446632
O -0.166414 -1.089749 1.605881
O 2.823074 -1.075261 -2.032364
O -2.501736 0.519719 0.141497
O 0.392133 1.435540 0.141522
O 3.066343 0.897250 -0.222209
H 1.330246 1.388271 -0.107845
H 0.406416 1.612679 1.083190
H -2.815583 0.515466 1.043755
H -2.487513 1.452933 -0.149840
H -1.006694 2.969053 -0.671177
H -2.291548 3.682614 -1.150728
H 3.343642 -1.471260 -2.726786
H 1.901710 -1.241572 -2.240228
H 0.690257 -0.778915 1.957490
H -0.376777 -1.930097 2.005368
H 3.760597 1.525646 -0.404167
H 3.122644 0.208669 -0.908252
O -2.256152 -2.321147 -0.117978
H -3.173003 -2.081451 -0.252591
H -2.129400 -3.155723 -0.567421
H 2.691601 0.290868 1.521194
O 2.117830 0.108536 2.275887
H 2.675124 0.007010 3.042560

5VIIIp
E = -1761.948660
O -2.063489 -1.374169 0.806629
Mn -0.318023 -0.285486 0.628028
O 0.264915 -1.401266 -1.117746
O -1.525749 1.173252 -0.262871
O 1.095123 0.882498 0.739718
O 0.242087 2.991343 -0.699432
O 2.857503 -1.205893 -1.512853
O -4.116014 -0.176447 -0.509025
O 3.965343 -0.024220 0.826124
H 1.943524 0.595446 1.070502
H -2.245001 -1.994182 1.507726
H -2.896402 -1.050457 0.425152
H 1.215907 -1.319743 -1.351999
H -0.022395 -2.273724 -1.371480
H -1.047483 1.991529 -0.535409
H -2.360725 1.080140 -0.716343
H 4.372146 -0.641213 1.431221
H 4.544596 0.734920 0.806818
H 3.324403 -0.757658 -0.799037
H 3.319973 -1.010271 -2.322650
H -4.828379 0.273692 -0.057220
H -4.513507 -0.585835 -1.276024
H 0.308762 3.887670 -0.383589
H 0.809236 2.435248 -0.140338

H 0.268185 -1.149184 1.847629

5VIIIq
E = -1761.949621
Mn 0.704696 -0.321143 -0.821345
H -0.864117 0.729718 -2.410298
O 1.142153 1.022194 0.714293
O 2.548286 -1.161423 -0.421982
O -0.334899 -1.727877 0.406187
H -0.249164 -2.676803 0.382439
H -1.299169 -1.502014 0.398033
O -2.668690 -0.768629 0.011640
H -2.416252 -0.027720 -0.540853
H -3.520583 -0.581292 0.423726
O -0.677646 0.752051 -1.476422
H 0.805823 -1.027055 -2.263414
H 1.885274 1.023983 1.315433
H 0.698438 1.897934 0.670532
O -0.250333 3.050141 -0.032002
H -0.653305 2.460686 -0.684492
H -0.921830 3.640074 0.296806
O 3.647032 -0.000374 1.780876
H 3.662068 -0.471082 2.613187
H 4.453448 0.513122 1.759057
H 3.087534 -0.856070 0.325411
H 3.085874 -1.655343 -1.035024
O -5.159423 -0.261565 1.093424
H -5.397729 -0.067641 1.996075
H -5.945564 -0.608077 0.678914

5VIIIr
E = -1761.946810
O 0.728083 0.463309 1.694110
Mn -0.580070 0.890912 0.007193
O 0.143586 2.819520 -0.270495
O -1.545961 -0.640228 -0.273702
O 1.984183 -1.874583 1.091062
O 0.258962 -2.388367 -0.965730
O -4.311108 -0.849899 0.191521
O 0.941956 0.262754 -1.284384
O 3.410389 0.079906 -0.366448
H -0.302669 3.626595 -0.023828
H 0.935140 3.039489 -0.758798
H 0.484119 0.619263 2.602826
H 1.197996 -0.388666 1.651077
H -2.486766 -0.700545 -0.082333
H -4.760009 -1.189178 0.961232
H -4.954567 -0.855464 -0.512300
H 0.773886 -0.646110 -1.568249
H 1.891118 0.286773 -1.043278
H 2.218305 -2.577272 1.692175
H 1.409817 -2.263422 0.408917
H 4.264653 0.035596 -0.787136
H 3.319980 -0.694851 0.195405
H 0.048089 -3.168512 -1.470749
H -0.583308 -1.949610 -0.712096
H -1.834739 1.573829 0.739837

5VIIIs
E = -1761.947065
O -1.640464 -0.940463 -1.509394
Mn -0.161859 -0.013393 -0.182031
O 1.185606 0.985014 1.239241
O 1.043996 -1.805138 -0.036717
O -1.529175 -0.726258 1.493050
O 3.572574 -1.041185 0.212665
O -3.774527 -0.004661 0.014058
O 3.006828 1.536919 -0.738662
H 1.947977 1.347247 0.762107
H -1.550966 -0.770205 -2.446101
H -2.560484 -0.784506 -1.262389
H 2.010641 -1.664589 0.029124
H 0.897478 -2.556226 -0.606911
H -1.398939 -1.528308 1.993675
H -2.466330 -0.663717 1.276978
H 3.516489 2.296066 -1.019279
H 2.276886 1.442733 -1.371032
H 3.683693 -0.195819 -0.235129
H 4.402273 -1.507732 0.163356
H -4.727810 -0.042108 0.051237
H -3.527391 0.921768 -0.016854
O -1.543330 1.839001 -0.018469
H -1.314591 2.386904 0.735190
H -1.231095 2.320540 -0.792794
H 1.516578 0.586942 2.043419

5VIIIt
E = -1761.943712
O 1.436365 1.354704 -0.944385
Mn -0.501682 1.003276 -0.342968
O -0.234850 2.246100 1.414301
O -2.202227 0.898817 0.273431
O 0.234098 -0.876620 0.339500
O -1.776091 -2.585212 0.434526
O -4.195220 -1.289806 -0.295528
H -2.906768 0.520331 -0.245916
H 2.045140 0.587624 -0.976748
H 1.624719 1.925063 -1.685756
H -0.444901 -1.574008 0.451528
H 1.062655 -1.262465 0.049858
H -1.078073 2.235607 1.869546
H 0.458296 2.417989 2.046169
H -4.680243 -1.607246 -1.054153
H -4.853230 -1.079007 0.363649
H -2.628464 -2.176039 0.243117
H -1.945216 -3.337329 0.993818
H -1.014318 1.185621 -1.854568
O 2.826741 -0.910929 -0.842692
H 2.995673 -1.423530 -1.630007
H 3.632122 -0.977344 -0.301104
O 5.056898 -1.099984 0.675193
H 5.880719 -0.636837 0.544672
H 5.228235 -1.776892 1.325347

7VIIIu
E = -1761.946549
O -2.397000 -1.462221 0.031139
Mn -0.749137 0.035236 -0.035985
O 0.642356 1.743936 0.095234
O 0.414016 -1.152651 1.411774

O -2.040031 1.001485 -1.538039
O 2.764258 -1.503912 0.032268
O 3.493577 1.225087 -0.039692
H 1.604546 1.643070 0.071121
H -2.292574 -2.387347 0.253947
H -3.048187 -1.408975 -0.670245
H 1.310888 -1.425502 1.172928
H 0.376063 -1.017796 2.356577
H -1.698464 1.180269 -2.426999
H -2.585483 1.766098 -1.305987
H 4.021639 1.636334 0.645489
H 3.850698 1.550134 -0.867491
H 3.229532 -0.657772 0.025636
H 3.426550 -2.192166 0.029246
O 0.451701 -1.106794 -1.487119
H 0.449025 -0.932487 -2.430053
H 1.343498 -1.382062 -1.234353
H 0.430609 2.500453 -0.462337
O -1.760829 0.955827 1.719576
H -1.563496 1.859046 1.975547
H -2.670815 0.781745 1.965462

5VIIIv
E = -1761.948544
O -1.519470 -1.521039 -0.543642
Mn 0.000183 -0.089442 0.041242
O 1.517874 -1.522567 -0.544584
O 1.481413 1.466144 -0.258687
O -1.480739 1.466911 -0.257742
O -3.905667 0.000965 -0.103635
O 3.905675 -0.002220 -0.103452
O 0.001885 3.759415 0.551626
H 1.289598 2.358244 0.043185
H 2.412185 1.262926 -0.129387
H -2.436384 -1.285267 -0.374078
H -1.345922 -2.396248 -0.182347
H -1.288259 2.359019 0.043561
H -2.411791 1.264597 -0.128974
H 2.435163 -1.287097 -0.376613
H 1.344822 -2.397742 -0.183175
H 4.398168 -0.084368 0.711675
H 4.560616 0.056888 -0.797355
H 0.002386 4.585766 0.071154
H 0.001977 3.997125 1.477685
H -4.557218 0.058698 -0.800847
H -4.402089 -0.079732 0.709238
O -0.001567 -3.483291 0.794730
H -0.000564 -3.064571 1.659191
H -0.002504 -4.426724 0.949123

5VIIIw
E = -1761.941921
O -1.813384 2.911819 0.396690
O -2.714753 0.513592 0.367139
Mn -0.975607 -0.374503 -0.486367
O -2.416121 -1.647594 -1.337801
O -0.622901 -1.625283 1.206363
O 0.053170 1.134165 -0.307299
O 1.763861 -1.055207 2.048433
O 2.883098 0.818905 0.352164
O 4.886996 -0.015274 -1.327540
H 1.009533 1.076508 -0.329655
H -2.970198 0.251290 1.249831
H -2.577373 1.495854 0.393093
H -0.948525 2.580028 0.105107
H -1.993450 3.733856 -0.049402
H 5.637714 -0.550624 -1.083659
H 0.258336 -1.447913 1.618708
H -0.793752 -2.560957 1.275376
H 3.184947 1.648187 0.714508
H 3.586605 0.521952 -0.244039
H -3.338617 -1.401463 -1.273739
H -2.296559 -2.184535 -2.118686
H 2.229390 -0.387486 1.522027
H 2.102639 -1.012101 2.937552
H 0.080034 -0.984935 -1.533559
H 5.059736 0.303929 -2.209472

5VIIIx
E = -1761.944676
O -2.391041 1.465354 0.017050
Mn -0.741926 -0.021640 0.025555
O 0.620199 -1.740478 -0.127440
O 0.421045 1.175687 -1.407350
O -2.015243 -1.019531 1.518905
O 2.770388 1.493252 -0.032043
O 3.461966 -1.242467 0.038682
H 1.582968 -1.641650 -0.111040
H -2.293115 2.396292 -0.181960
H -3.021021 1.391855 0.735733
H 1.319888 1.439042 -1.162977
H 0.379611 1.072020 -2.355711
H -1.642986 -1.227494 2.389443
H -2.571862 -1.774724 1.283804
H 4.010470 -1.664664 -0.623657
H 3.781204 -1.568938 0.881768
H 3.228062 0.642699 -0.029394
H 3.438588 2.175758 -0.020955
O 0.463313 1.081617 1.491979
H 0.477830 0.847978 2.422400
H 1.354304 1.360262 1.239727
H 0.413075 -2.478410 0.457068
O -1.785270 -0.941281 -1.704074
H -1.577308 -1.837047 -1.976104
H -2.694407 -0.769188 -1.952697

5VIIIy
E = -1761.947254
O 1.721662 -1.953735 0.293238
Mn 0.171367 -0.425890 0.102187
O 1.768404 0.945029 -0.230553
O -1.316737 1.241458 -0.280863
O -1.079603 -1.570133 -1.237868
O -3.546112 -0.682763 -0.710008
O 4.160995 -0.548315 0.261216
O -2.814118 0.269497 1.824213
H -1.880111 1.214513 0.508849
H -1.919168 1.063419 -1.006448
H 1.593097 -2.573347 1.010771
H 2.639416 -1.652674 0.330391

H -2.041102 -1.446390 -1.133455
H -0.915175 -2.460075 -1.539870
H 1.610214 1.901629 -0.308035
H 2.696480 0.762401 -0.075274
H -3.237198 0.578044 2.624466
H -2.029513 -0.232454 2.098792
H -3.611008 -0.505665 0.235730
H -4.413071 -0.937908 -1.014650
H 4.793332 -0.700137 -0.439576
H 4.680172 -0.425571 1.054548
O 0.740443 3.378724 -0.385395
H 0.857854 4.263460 -0.718818
H -0.187856 3.159902 -0.453926

IR frequencies (in cm^{-1}, without shift) and intensities (KM/mole) for structures optimized at the BHandHLYP/aug-cc-pVDZ level

7IIa
30.7313 1.5476
87.9991 0.8598
236.9107 2.3411
276.1865 20.9571
304.3383 4.7
345.2992 225.8488
385.3786 226.5671
484.8917 16.0658
491.3515 60.4095
1679.5964 125.2825
1689.8342 104.4727
3883.4468 87.7403
3891.2236 60.3704
3974.4079 186.5041
3989.0069 189.9172

7IIb
64.4194 0.4304
150.5894 17.4743
259.569 66.7381
284.6552 240.7681
287.465 104.8601
356.195 9.1429
425.3049 29.3894
677.675 16.1289
958.016 116.8184
1671.4813 110.3169
1712.0438 24.4526
3262.5427 1644.2576
3940.0512 76.9068
3953.7438 143.747
4037.8105 168.9853

5IIc
33.2178 0.0
84.9665 11.5522
85.5225 15.9741
260.9294 0.0
264.1715 401.5665
326.3575 0.0
438.8574 15.2654
513.9253 8.953
518.7483 0.0
1672.7824 293.1942
1675.6326 0.0
3888.8677 0.0
3889.1754 180.4236
3982.5449 0.0
3983.862 432.272

5IId
72.5571 0.5696
142.1434 17.5368
266.7639 235.5138
272.7132 91.5338
275.9713 61.8532
362.6967 14.4853
469.074 8.3496
709.2875 14.1721
979.872 105.0031
1671.0543 118.7193
1707.7235 31.6763
3188.5029 1851.9349
3939.3482 78.4426
3955.0165 162.7554
4037.4487 172.1753

5IIe
108.1392 8.1895
127.2444 1.6808
161.2034 14.1344
244.1491 95.7888
376.4654 316.9314
386.8627 4.8363
471.2303 21.7081
612.767 55.9616
648.012 95.7626
755.4344 60.3741
1518.7647 40.5962
1696.1539 125.873
3876.0383 226.9629
3958.9199 273.9858
4001.3882 312.5054

7IIIa
73.8619 1.2155
74.3209 1.2152
91.1358 0.5686
180.6567 4.042
180.8 4.0485
263.1754 5.1014
263.2897 5.1167
287.7604 1.3682
300.0963 7.1787

374.2774 295.3887
377.5165 151.9465
377.8763 151.7147
469.8298 15.7541
469.9987 15.7772
495.4699 133.0868
1677.694 157.9717
1677.7737 157.7355
1678.8535 50.3446
3897.8261 83.9487
3897.8793 84.0586
3898.4833 7.7427
3995.5277 143.954
3995.8322 174.1667
3995.8998 175.0731

5IIIb
39.9443 0.2566
137.7076 6.5883
138.6667 1.0098
186.3595 7.7116
199.8761 22.2406
278.438 121.1408
351.2413 17.6098
353.5917 42.8807
389.0305 2.5379
397.9657 345.9156
441.6536 152.9358
535.3992 6.2833
609.2993 125.3687
663.5125 41.6758
703.1783 134.2542
766.7498 17.9313
1637.7216 4.1844
1665.662 178.7603
1693.3528 105.9689
3877.4948 140.5071
3905.7061 165.3609
3995.901 178.3021
4004.1679 269.4672
4014.6052 202.3489

7IVa
42.9684 7.521
45.1123 4.9006
94.2161 0.4003
122.5136 12.6196
131.4714 1.4298
137.6719 0.0516
222.6715 59.7529
230.882 24.579
264.9511 2.426
272.371 1.2068
273.2011 12.35
282.0133 0.0701
297.8947 160.971
305.7793 122.5881
312.9332 71.9995
455.5254 116.4886
455.7145 40.8246

542.6105 121.0306
624.3038 179.7915
650.3196 11.4179
723.8763 135.2067
1665.4592 122.4864
1671.4835 162.6954
1676.7 108.9425
1714.6401 0.9317
3732.7415 56.004
3769.5023 806.5082
3906.337 26.8442
3922.0913 43.383
3987.7632 288.3263
3990.3348 68.3337
4010.9912 139.9135
4011.7803 178.9922

7IVb
19.5168 3.0178
42.6985 1.336
69.557 1.3089
78.5286 0.3916
98.9587 1.6959
121.5735 6.0782
149.4596 11.4349
213.2874 8.5387
247.9119 29.9935
263.5666 22.7448
268.5113 192.3799
282.9537 28.0317
310.0129 168.2474
321.2488 60.7979
340.662 109.935
361.3521 14.209
374.4941 151.0492
460.8868 29.7108
477.1243 48.7172
617.9543 72.3425
881.2137 102.5294
1671.4088 135.491
1673.4047 118.7798
1677.6679 132.9661
1711.997 42.9563
3499.9625 1132.5826
3904.6508 53.0451
3907.5688 39.7513
3946.2533 50.8363
3976.4224 128.7237
4005.4004 157.5912
4010.2682 153.7353
4044.3099 145.4817

7IVc
54.0779 5.4163
64.9725 1.0025
74.0696 0.1122
80.0936 0.8656
98.9242 1.2561
152.5145 0.5326
176.223 1.778

184.1201 5.9933
198.6998 0.2392
248.8043 12.6683
255.1553 0.385
282.1176 194.8212
290.2213 2.7854
366.2371 0.2549
373.7828 218.3584
380.3769 51.4967
387.6279 152.2468
409.6778 35.2781
432.7073 132.936
467.785 218.3058
508.0248 25.6996
1650.5751 130.042
1667.7385 125.1579
1678.4559 217.9946
1679.594 13.9199
3898.6871 35.2602
3898.8526 37.6285
3905.3939 19.577
3917.3026 60.68
3997.4311 30.3578
3997.6579 207.6406
4011.8939 154.2561
4024.1055 147.0971

7IVd
43.5677 1.8151
46.0031 0.8551
76.8039 7.9126
115.2282 0.6609
121.8842 0.9161
131.304 1.0686
203.3622 129.171
216.0635 151.7201
261.7982 33.7986
263.1669 0.0007
265.7158 59.9836
303.004 55.566
312.6232 16.5709
324.8286 20.2735
400.5175 22.186
464.4597 158.1741
601.1006 122.2547
630.1918 37.1428
733.7736 170.7535
750.6448 83.1769
882.8722 93.2025
1663.8138 125.3614
1679.1095 94.8195
1681.3577 85.5834
1714.1317 1.4444
3498.5837 1309.5113
3693.5433 453.2755
3833.0903 591.7939
3924.8967 31.2302
3952.0663 48.3787
3989.8578 188.0712
4014.1241 154.1246
4052.3723 148.634

5IVe
67.3988 3.9164
76.2926 7.6453
92.2267 1.0046
109.2214 1.0926
168.0119 1.0583
174.7699 8.5842
224.3427 97.4097
231.9229 23.2782
282.0973 37.2427
302.036 1.0713
319.4474 22.9073
345.656 133.3019
356.7796 24.0056
374.9136 84.849
415.0821 152.1158
457.329 389.9039
497.9115 30.2685
539.1565 3.0702
591.7115 138.4476
664.015 79.3901
667.5775 92.554
757.4491 32.1734
1610.4642 4.1794
1671.3643 158.6301
1676.2725 142.3698
1686.9227 89.0073
3880.937 105.2335
3914.9822 178.0841
3920.0775 67.8628
4002.586 141.9144
4005.9328 249.0783
4021.6249 170.3057
4026.9378 167.4577

5IVk
57.2412 1.3171
104.9497 2.4788
109.8402 2.6424
143.8706 2.6316
176.2974 8.5456
240.3235 11.2215
248.2196 6.1937
259.2606 20.2035
310.534 3.4907
351.1647 1.0521
370.4094 27.6365
386.8653 101.7293
395.05 352.2192
420.6617 2.9036
500.5231 159.1732
599.5056 58.292
609.5015 58.6038
644.7412 88.9177
709.2615 205.5215
749.2148 107.8348
765.3785 3.0502
807.5329 134.966

1635.0347 156.1902
1669.5699 91.1796
1677.2874 27.7072
1710.8261 28.7082
3587.9117 840.1542
3836.9848 414.7258
3914.4335 49.6618
3983.313 352.172
3991.2307 169.6698
4000.8507 173.8603
4020.4687 186.0336

5VIIIa
56.1836 2.8279
82.6794 5.4571
87.9952 14.8989
106.2764 7.797
113.3035 2.2404
126.3205 0.6609
128.4093 12.6439
143.9006 1.8382
155.1619 10.4963
161.7271 4.6362
169.3527 4.942
179.0415 48.1226
195.0302 1.9755
216.0418 16.9606
219.8302 8.4345
227.0355 1.1904
250.6329 30.9867
252.6946 34.3378
278.5071 96.6289
282.6287 117.9877
292.4655 162.0951
306.7478 21.5988
322.0039 112.5764
346.3223 14.6932
349.3986 73.9521
362.629 66.053
381.7001 4.9279
399.0197 7.3065
412.8615 26.4834
458.1746 28.1758
487.4583 39.3553
523.0303 95.9834
527.9529 72.4698
562.042 75.8904
576.6586 66.3455
596.1288 39.2398
605.9842 162.9757
678.6118 38.224
705.8103 41.4854
716.2364 186.1111
765.7011 293.1426
772.6479 201.3302
842.6164 131.838
879.7445 8.5656
893.2001 241.7534
981.8891 108.5766
1584.9211 2.0955
1666.3969 154.2736
1672.7178 97.6012
1679.1139 63.1504
1691.4153 148.3531
1706.6055 25.8679
1719.4231 123.6202
1756.0382 3.02
3263.8591 1140.1113
3639.4228 99.5799
3666.9856 536.2394
3701.6622 412.628
3720.4376 648.2642
3768.7657 717.7275
3790.3517 512.4891
3933.6497 112.1425
4006.911 118.1358
4009.5589 143.8751
4012.7624 184.7434
4015.1264 137.8565
4020.4195 127.1172
4039.7758 82.8961
4041.58 202.778

5VIIIb
24.6509 4.6295
32.816 1.3058
46.9682 6.5104
85.677 0.6108
88.305 0.2568
105.8093 7.5061
122.5486 1.9171
135.1976 4.2449
136.6115 17.6364
151.1103 3.7234
158.3863 3.5737
181.1201 5.6078
208.2822 32.1122
230.3806 13.4886
234.7202 0.5505
237.2547 58.992
240.5538 12.6553
251.8278 15.055
275.7656 36.3359
282.1142 9.3316
292.9464 26.3339
295.4825 28.9259
306.961 118.1909
332.0264 134.7602
339.5503 9.5381
363.9937 29.9761
370.1719 72.052
377.8102 97.2409
390.5086 49.7058
439.5386 138.4563
457.5497 68.4844
497.0016 27.7368
525.6935 116.8628
550.2326 20.039
580.3709 92.7639
600.6034 98.3965

608.1459 108.8684
650.4264 244.3161
669.0706 146.9288
678.7387 58.0937
719.2836 48.2575
762.1639 340.4142
772.6401 70.8754
831.7496 57.8828
892.402 151.705
916.1405 100.6707
1532.941 4.2801
1651.5175 142.6476
1666.4653 131.274
1679.3864 112.9561
1686.9022 110.3506
1700.1356 9.3546
1710.1721 55.642
1735.6778 8.8926
3424.9757 517.8391
3522.8403 1213.7644
3609.1223 164.1676
3680.3065 1238.1021
3751.5199 449.0398
3849.6718 438.2563
3930.2528 30.709
3988.3653 151.8651
4002.6796 136.2131
4014.2374 276.8231
4018.6125 111.2953
4018.7034 134.0431
4021.5641 153.009
4035.1279 56.852
4036.4225 167.2069

5VIIIc
39.6587 1.609
56.5261 0.7463
81.834 0.6691
91.906 1.0915
107.8239 1.5161
121.2578 2.5581
122.5186 6.3441
142.6608 4.3271
154.3473 8.4543
165.9881 1.7434
181.6984 9.8226
190.9149 23.3275
201.5171 52.7815
222.3525 20.4409
224.677 49.2845
244.8384 11.852
253.6508 58.946
262.6288 15.8096
290.3291 44.5555
299.0909 30.8619
306.6568 22.3265
322.4146 10.2702
350.3973 29.8699
371.9556 15.0292
383.5327 230.8236
388.4425 42.6057
401.2526 52.0097
433.3302 41.8736
439.706 8.0419
488.1681 41.3443
518.6282 18.1986
536.8558 147.9469
574.3558 100.5573
610.4692 87.474
620.9477 97.5077
635.569 103.5312
668.7563 142.2984
710.3452 16.3521
732.5053 109.1378
753.1629 313.3511
764.2039 77.3832
832.6377 141.0305
843.5319 178.8427
887.6251 141.5397
899.6607 109.1641
941.9603 47.8145
1630.9286 1.603
1678.1316 139.2241
1685.3539 104.2739
1695.2328 48.705
1706.4088 93.0063
1716.0331 17.6517
1727.6257 106.482
1748.8589 24.2426
3277.4207 1290.642
3437.3637 750.6432
3592.3905 493.9691
3647.7142 323.1822
3696.8005 829.1636
3744.4869 476.9955
3768.0862 998.8307
3814.0506 265.6667
4001.1116 135.7891
4003.7825 159.764
4010.5116 155.1543
4013.706 164.4214
4015.9273 124.7526
4024.406 198.9524
4040.3956 111.9448

5VIIId
22.732 1.6033
30.84 0.2241
47.5509 2.3155
54.7691 1.1247
69.8367 4.3644
77.6998 4.3079
100.0191 3.1407
106.7883 4.8337
130.211 1.7509
139.7694 7.6474
153.9238 7.9354
161.0929 7.8739
168.7724 213.3425
183.3779 3.667

212.3145 6.209
219.5235 68.502
237.4034 40.3167
252.116 12.5469
260.6788 32.5974
273.6764 4.4652
279.449 87.509
288.3607 46.629
306.9081 11.5836
314.6349 17.6027
330.3155 177.0222
350.6962 7.0456
365.4091 34.5248
374.7828 204.9829
385.7183 8.8052
411.4814 14.4445
420.5612 75.7705
462.788 198.7232
487.4739 18.2222
506.5942 55.2512
543.9267 5.3379
583.2721 72.9249
607.6409 113.7286
648.4915 153.9599
665.3655 107.4823
710.0712 94.0263
740.9246 297.4551
762.5138 36.0303
827.3995 31.6383
875.9369 184.294
900.0721 114.7147
938.5283 87.5473
1592.5038 1.3597
1649.3033 141.0149
1669.1627 92.1325
1678.5156 87.988
1682.1992 107.753
1694.0524 123.6328
1705.1637 48.7566
1729.4687 5.392
3431.2623 521.4472
3520.5508 1695.3061
3551.4631 839.2745
3584.8107 436.9726
3694.5882 892.4879
3921.5976 57.1296
3956.0459 53.3899
3991.1116 164.8389
3991.8684 145.3535
4013.2904 152.9969
4018.4653 105.6079
4031.1868 72.4398
4036.1338 277.5393
4036.7772 21.7641
4057.2645 151.0966

5VIIIe
28.5206 0.1259
44.272 0.6554
64.7363 13.4584
78.6248 0.0536
88.1374 0.2866
108.1373 1.5141
115.5768 3.934
127.8249 4.8666
149.5447 3.8025
151.526 4.1923
169.4226 6.0828
182.8688 2.6347
198.4364 125.787
206.0648 51.5012
215.5309 50.9608
237.572 13.8914
239.9132 34.8144
272.4414 46.2683
278.922 14.5741
292.0876 118.7396
302.2137 62.031
316.1474 24.5717
318.2483 17.7465
332.9461 17.0885
365.9241 51.8961
368.2267 62.8188
384.964 89.3352
396.9832 30.4865
430.8961 112.5027
434.8346 348.3364
468.3361 10.1053
487.3263 34.1793
530.9574 35.5362
541.6015 64.2345
567.863 15.7021
593.856 145.954
616.2489 18.0209
656.9221 259.4174
692.3595 32.9955
733.201 270.7121
737.8507 148.1251
761.2398 37.4377
838.6419 36.2401
873.2222 170.3138
893.1063 112.2406
937.7101 75.5455
1516.8739 12.5315
1642.4099 80.9804
1661.0224 98.1885
1674.0572 148.9232
1681.9538 135.211
1697.0507 68.365
1702.3612 53.4237
1726.4131 5.0126
3445.1087 332.5328
3470.1905 1030.9306
3574.5906 522.5168
3591.1992 891.9697
3687.6788 983.031
3754.6562 448.862
3926.448 101.5559
3997.4647 194.926
4005.1914 48.3467

4009.0102 169.4377
4015.2688 108.7967
4018.9573 139.5471
4025.5714 183.5996
4034.4058 124.3278
4038.856 195.6519

5VIIIg
19.1493 5.0972
29.792 1.06
35.96 4.9159
41.7009 0.693
53.3881 2.1232
57.6729 0.4791
80.3761 0.8264
97.4741 7.5928
107.4439 1.7912
109.2036 0.6733
122.1422 1.1444
127.1349 6.5156
153.785 1.1768
155.286 68.8678
173.8331 4.8784
189.9604 71.7761
192.6165 26.332
202.8694 162.8109
214.337 65.0791
218.8506 77.4018
230.6922 46.6529
263.7473 9.0374
270.2147 10.7719
277.5581 19.2261
302.6749 18.7495
316.0735 6.9734
328.6444 35.0115
349.8983 27.4359
377.6002 12.4494
388.0885 54.1246
398.1797 13.3936
444.0543 136.8775
456.6165 74.7829
523.0103 158.5969
558.1364 74.1446
608.1108 42.0591
620.7414 141.878
622.0042 66.4018
645.81 53.3116
699.0647 226.8559
704.6638 155.2827
784.131 49.4275
811.6154 152.3909
816.6266 167.201
881.7702 47.4243
1670.1691 125.7279
1672.7373 18.1924
1678.2198 204.15
1682.1804 107.6207
1695.5989 62.6176
1697.021 39.3709
1713.9447 16.3507
1721.3673 12.6427
3562.8917 965.8073
3628.1143 976.5264
3643.0297 344.9133
3720.9604 523.4102
3744.8127 231.8459
3814.2385 813.5149
3833.5044 228.8297
3851.3615 683.6023
3930.8797 10.5185
3957.1525 35.2266
4002.9724 100.7719
4009.14 195.7355
4016.8027 90.7186
4021.7775 147.0779
4032.1818 177.2857
4058.9382 129.7427

References

1 A. Akhgarnusch, W. K. Tang, H. Zhang, C.-K. Siu and M. K. Beyer, *Phys. Chem. Chem. Phys.*, 2016, **18**, 23528.
2 M. Allemann, H. Kellerhals and K. P. Wanczek, *Int. J. Mass Spectrom. Ion Process.*, 1983, **46**, 139.
3 C. Berg, T. Schindler, G. Niedner-Schatteburg and V. E. Bondybey, *J. Chem. Phys.*, 1995, **102**, 4870.
4 T. Schindler, C. Berg, G. Niedner-Schatteburg and V. E. Bondybey, *Chem. Phys.*, 1995, **201**, 491.
5 A. Akhgarnusch, R. F. Höckendorf and M. K. Beyer, *J. Phys. Chem. A*, 2015, **119**, 9978.
6 P. Caravatti and M. Allemann, *Org. Mass Spectrom.*, 1991, **26**, 514.
7 V. E. Bondybey and J. H. English, *J. Chem. Phys.*, 1981, **74**, 6978.
8 T. G. Dietz, M. A. Duncan, D. E. Powers and R. E. Smalley, *J. Chem. Phys.*, 1981, **74**, 6511.
9 A. G. Marshall, C. L. Hendrickson and G. S. Jackson, *Mass Spectrom. Rev.*, 1998, **17**, 1.
10 R. L. Wong, K. Paech and E. R. Williams, *Int. J. Mass Spectrom.*, 2004, **232**, 59.
11 O. P. Balaj, C. B. Berg, S. J. Reitmeier, V. E. Bondybey and M. K. Beyer, *Int. J. Mass Spectrom.*, 2009, **279**, 5.
12 D. Thölmann, D. S. Tonner and T. B. McMahon, *J. Phys. Chem.*, 1994, **98**, 2002.
13 R. C. Dunbar, *Mass Spectrom. Rev.*, 2004, **23**, 127.
14 T. Schindler, C. Berg, G. Niedner-Schatteburg and V. E. Bondybey, *Chem. Phys. Lett.*, 1996, **250**, 301.
15 P. D. Schnier, W. D. Price, R. A. Jockusch and E. R. Williams, *J. Am. Chem. Soc.*, 1996, **118**, 7178.
16 M. Sena and J. M. Riveros, *Rapid Commun. Mass Spectrom.*, 1994, **8**, 1031.
17 B. S. Fox, M. K. Beyer and V. E. Bondybey, *J. Phys. Chem. A*, 2001, **105**, 6386.
18 A. Herburger, C. van der Linde and M. K. Beyer, *Phys. Chem. Chem. Phys.*, 2017, **19**, 10786.
19 W. A. Donald, R. D. Leib, M. Demireva and E. R. Williams, *J. Am. Chem. Soc.*, 2011, **133**, 18940.
20 A. Herburger, M. Ončák, C.-K. Siu, E. G. Demissie, J. Heller, W. K. Tang and M. K. Beyer, *Chem. Eur. J.*, 2019, **25**, 10165.
